# Potential Benefits of Cattle Vaccination as a Supplementary Control for Bovine Tuberculosis

**DOI:** 10.1371/journal.pcbi.1004038

**Published:** 2015-02-19

**Authors:** Andrew J. K. Conlan, Ellen Brooks Pollock, Trevelyan J. McKinley, Andrew P. Mitchell, Gareth J. Jones, Martin Vordermeier, James L. N. Wood

**Affiliations:** 1 Disease Dynamics Unit (DDU), Department of Veterinary Medicine, University of Cambridge, Cambridge, United Kingdom; 2 Data Systems Group, Animal & Plant Health Agency Weybridge, New Haw, Addlestone, Surrey, United Kingdom; 3 Department of Bacteriology (TB Research), Animal & Plant Health Agency Weybridge, New Haw, Addlestone, Surrey, United Kingdom; The Pennsylvania State University, UNITED STATES

## Abstract

Vaccination for the control of bovine tuberculosis (bTB) in cattle is not currently used within any international control program, and is illegal within the EU. Candidate vaccines, based upon Mycobacterium bovis bacillus Calmette-Guérin (BCG) all interfere with the action of the tuberculin skin test, which is used to determine if animals, herds and countries are officially bTB-free. New diagnostic tests that Differentiate Infected from Vaccinated Animals (DIVA) offer the potential to introduce vaccination within existing eradication programs. We use within-herd transmission models estimated from historical data from Great Britain (GB) to explore the feasibility of such supplemental use of vaccination. The economic impact of bovine Tuberculosis for farmers is dominated by the costs associated with testing, and associated restrictions on animal movements. Farmers’ willingness to adopt vaccination will require vaccination to not only reduce the burden of infection, but also the risk of restrictions being imposed. We find that, under the intensive sequence of testing in GB, it is the specificity of the DIVA test, rather than the sensitivity, that is the greatest barrier to see a herd level benefit of vaccination. The potential negative effects of vaccination could be mitigated through relaxation of testing. However, this could potentially increase the hidden burden of infection within Officially TB Free herds. Using our models, we explore the range of the DIVA test characteristics necessary to see a protective herd level benefit of vaccination. We estimate that a DIVA specificity of at least 99.85% and sensitivity of >40% is required to see a protective benefit of vaccination with no increase in the risk of missed infection. Data from experimentally infected animals suggest that this target specificity could be achieved in vaccinates using a cocktail of three DIVA antigens while maintaining a sensitivity of 73.3% (95%CI: 61.9, 82.9%) relative to post-mortem detection.

## Author Summary

Bovine tuberculosis (bTB) is a major economic disease of livestock worldwide. Despite an intensive, and costly, control program in the United Kingdom, bTB continues to persist. Vaccination can provide some protection to cattle, but is currently illegal within the European Union due to the interaction of BCG with the action of the tuberculin skin test. The EU has signaled that changes in legislation will require field validation of BCG as a supplement to existing controls. A particular concern is that the imperfect sensitivity of prospective DIVA tests for vaccinates may increase the chances of infection being missed within herds. However, we demonstrate that high DIVA specificity will also be essential in order for farmers to see a protective herd level benefit of vaccination in terms of the frequency of tests they are subjected to and number of animals condemned. Field validation of the DIVA test will be an essential prerequisite to use of BCG in the field. Our estimated target specificity provides an important criterion for validation of prospective DIVA tests before deployment in the field.

## Introduction

Human vaccine *Mycobacterium bovis* bacillus Calmette-Guérin (BCG) vaccination has been shown to induce significant levels of protection in cattle in a large number of experimental studies and field trials since 1912 (reviewed in [1]). More recent laboratory studies have demonstrated significant reductions in the development and presentation of visible lesions after challenge with *M*. *bovis* [2]. Recent field trials and experiments under natural transmission conditions have also reported its effectiveness, although with variable levels of protection [3], [4] ranging from no effect to up to individual protective efficacies of 68% [5]. However, vaccination is not currently used as part of a national bovine TB control program. By far the most important historical barrier to the use of cattle vaccination is the interference of BCG with the specificity of the tuberculin skin test [6], which is the cornerstone of surveillance and eradication strategies [7]. A new generation of diagnostic DIVA tests that can Differentiate Vaccinated from Infected Animals [8], [9] opens up the opportunity for the use of BCG within current control programs. For vaccination to be feasible economically and useful within the context of European legislation, the benefits of vaccination must be great enough to outweigh any increase in testing associated with the efficiency of DIVA testing. In this study we use rigorously estimated within-herd transmission models [7] to explore scenarios for the supplemental use of BCG vaccination in Great Britain. We estimate the DIVA test characteristics necessary to see a protective herd level benefit of vaccination when used within the current statutory system of testing.

Control of *M*. *bovis* infection of cattle in Great Britain, and internationally, generally depends on active disease surveillance through repeated testing of herds using tuberculin. The sequence of testing used has been designed around the known imperfect sensitivity and specificity of the tuberculin test. A fundamental challenge to the evaluation of diagnostic tests for bTB in GB is that test-positive animals are slaughtered irrespective of development of physical symptoms of disease. In the absence of a gold standard, the sensitivity and specificity of diagnostic tests can only be directly measured relative to culture confirmation of visible lesions. In this paper, unless otherwise stated, we define (and estimate) the sensitivity and specificity of diagnostic tests relative to the true infection status of animals. In our modelling framework we therefore define the specificity of a test as the complement (1-p_FP_) of the probability of obtaining a false-positive test result (p_FP_).

In the UK, the single Intra-dermal Cervical Comparative Tuberculin (SICCT) test is employed, which controls for cross-reactivity resulting from exposure to other related environmental mycobacteria. Animals are classified as test positive, or reactors, when the difference in a hypersensitivity reaction to bovine and avian derived tuberculin exceeds a fixed threshold. The level of this threshold is adjusted to calibrate the sensitivity and specificity of the SICCT test based upon the testing history of a herd. Under the standard interpretation used for routine testing in Great Britain, the SICCT test has an exceedingly high estimated specificity of > 99.99% [10] and a sensitivity relative to visible lesions with variable estimates of between 55.1%–95.5% [11], [12].

Once infection is detected within a British herd, movement restrictions are applied and all bovines within the herd must undergo a statutory sequence of (60 day) short interval testing. Described as a herd ‘breakdown’, provided that no animals are found to have visible lesions, the herd is classified as Officially TB-free Suspended (OTF-S) and restrictions are maintained until the herd passes at least one whole herd test at the standard interpretation [7]. However, when animals are found with lesions visible at post-mortem, or *M*. *bovis* is cultured from them, the herd is classified as Officially TB-free Withdrawn (OTF-W) and a more severe interpretation of the test is used, trading off specificity to increase relative test sensitivity with estimates between 88.5% and 100% [11], [12]. Since laboratory confirmation of lesions is itself insensitive, the true sensitivity of tuberculin testing may be considerably lower than these estimates as suggested by recent non-gold standard latent class analyses [13]. Inherent in the design of the sequence of testing using in GB is the desire to optimize the removal of infected animals by an insensitive, but highly specific, diagnostic test.

**Fig 1:**
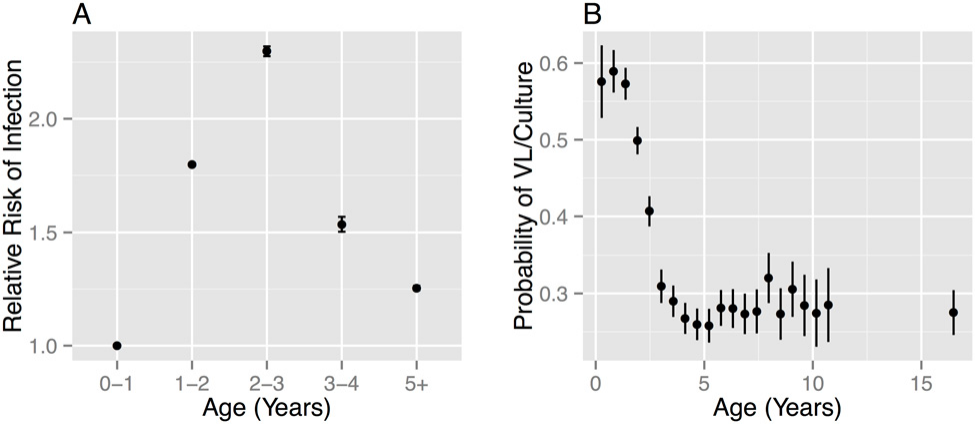
Age-stratified patterns of reactors in Great Britain. **A** The relative risk of infection by age RR(a) measured relative to the risk for 0–1 year old cattle, Error bars denote 95% credible intervals. RR(a) is calculated as the ratio of the force of infection for each age group divided by the estimate for the 0–1 year old group. The force of infection is a combined estimate for all national herds (beef and dairy) calculated from reactor cattle reported between 2004–2009 as described in [21]. **B** The probability of “confirmation” of reactor animals by the presence of visible lesions or culture as a function of age at slaughter, estimated as the proportion of reactor animals within each (200 day) age-class that demonstrated visible lesions or a positive culture result. Estimates are calculated using all reactor animals from within our study population of herds. The qualitative pattern is robust between different test-types and parish testing intervals [21].

In a previous study of the within-herd persistence of bTB we set out to quantify the contribution that these imperfections of tuberculin testing have on the within-herd persistence of infection [7]. We estimated that up to 50% of herds had a median residual burden of infection of one animal remaining when movement restrictions were lifted. We also found that rates of recurrence were primarily driven by the rate of re-introduction of infection into the herd, especially in high incidence regions. However, the time for a herd to clear restrictions was remarkably consistent between low and high incidence areas (Fig. 1), with herd size and use of the severe interpretation of the SICCT test being the only clear risk factors associated with the duration of restrictions [14]. Taken together, these observations suggest that SICCT specificity is an important determinant of the time for herds to clear restrictions under the current regulatory regime.

The duration of such movement restrictions is important due to the considerable economic burden they place on farms. The financial costs to farmers caused by breakdown restrictions vary massively, depending on a complex range of factors including the farming business model, the length of restrictions, timing of the breakdown and the individual animals affected [15]. It is more straightforward to quantify the costs to government, which depend on the number of visits to farms by veterinarians, tests carried out and compensation for animals condemned as reactors, all of which have increased with the growing prevalence of infection over the last two decades [16]; costs are estimated to be up to £0.5 billion pounds over the last ten years [17].

Due to the indirect protection afforded by vaccination through herd immunity [18], vaccination is often the most cost-effective method of controlling infectious diseases. Cattle vaccination has been proposed as a supplementary method to reduce the duration of time that herds are under restrictions and the number of animals removed during testing. However, such use poses a particular challenge for *M*. *bovis* infection where the known interference of BCG with tuberculin testing has resulted in cattle vaccination for bTB being prohibited in the EU. Tuberculin testing is used to demonstrate progress towards national eradication and also as the basis of international trade in cattle. Thus, vaccinated animals that demonstrate sensitivity to tuberculin have to be treated as infected animals and slaughtered.

There has recently been movement in this position by the EU and requirements for changes in legislation to allow cattle vaccination have been outlined, essentially requiring field evaluation of both BCG efficacy and DIVA test characteristics. Recent advice from the European Food Safety Authority [19], commissioned by the EU, emphasized the importance of demonstrating that BCG is efficacious and that DIVA tests can be shown to have a comparable sensitivity to tuberculin testing. However, a key factor overlooked in this report was that the currently viable DIVA tests are based on the gamma-interferon platform, which is known to have a lower specificity than SICCT testing [8]. This raises the concern that the use of BCG vaccination and DIVA tests under the current regulatory system of testing may lead to vaccinated herds effectively unable to escape restrictions once a single reactor animal has been detected.

Here, we extend our previous work [7], incorporating a more realistic herd demography, essential for assessing the impact of vaccination, into our rigorously estimated within-herd models of bTB transmission. We explore scenarios for the use of BCG vaccination in combination with DIVA testing and consider the potential economic benefits relative to the current regime of tuberculin testing. We wish to explore the potential for economic benefits not only from the perspective of the government, which currently pays for testing and compensation for test-positive animal but also the individual farmer. Studies of the financial costs to farmers associated with bTB restrictions have shown that the financial outcomes of a bTB incident are complex and highly variable for different farms to the extent that they cannot be summarized through averages or point estimates. From the perspective of simulation this variability in financial costs is dependent on specific aspects of farm management and economic variables that have no meaningful analogues within our epidemiological models [15].

In the interest of transparency we therefore choose to use indirect proxy measures of economic costs based on the probability of restrictions being applied to a herd, the number of tests and animals condemned as reactors and thus the duration of time that herds are under restrictions. The most important of these proxy measures is the risk of restrictions being applied and subsequently recurring after they have been lifted. In a study of farmer’s attitudes and willingness to pay for cattle vaccination, Bennett and Balcombe found that farmers valued reductions in the risk of restrictions being applied over the severity of an incident in terms of duration or animals lost [20]. As such we would contend that reducing the risk of restrictions being applied to herds is the single most important requirement for cattle vaccination to be accepted and adopted by farmers.

We demonstrate that DIVA specificity, rather than sensitivity or individual level efficacy, is the dominant factor determining whether BCG vaccination can provide a protective economic benefit at the herd level when used as a supplement to existing controls. We explore the extent to which derogations on the requirements of testing can be used to mitigate the negative effects of DIVA testing and estimate break-even test characteristics in terms of these key epidemiological and economic measures of costs.

## Results

### Age-structured model

The compartmental models developed in [7] assumed that the movement of animals on and off a herd occurred at constant herd level turnover rate sampled from the Cattle Tracing System (CTS). As a consequence of this approximation, individuals spend an exponentially distributed duration of time on herds. In practice the distribution of residence times on herds can vary considerably between different business models impacting on the herd level rates of removal of residual infection from herds. Implementing these models within an individual based framework allows us to model more realistic residency times of individuals on herds and more accurately capture the variation in turnover rates between herds. An additional benefit of the individual based model framework is that we can also incorporate new evidence for the relative risk of infection with age and probability of reactor animals demonstrating visible lesions recently estimated by [21] (Fig. 1). After adjusting for testing patterns, we found that young beef and dairy animals experience a similar infection risk that peaks at 36 months before falling and plateauing for animals older than five years (Fig. 1A, Table 1). Although the infection risk is lowest in cattle under 12 months, the highest proportion of skin test positive animals is found to have visible lesions when they are examined at slaughter (Fig. 1B, Table 2).

**Table 1:** Relative Risk of Infection RR(a).

Age Range(Years)	Relative Risk
0–1	1.0
1–2	1.8
2–3	2.3
3–4	1.5
4+	1.3

**Table 2:** Age stratified probability of reactors with visible lesions/culture positive (*P*_*VL*_(*ɑ*)).

Age Range (Days)	Probability of VL/Culture
0–200	0.58
200–400	0.59
400–600	0.57
600–800	0.50
800–1000	0.41
1000–1200	0.31
1200–1400	0.29
1400–1600	0.27
1600–1800	0.26
1800–2000	0.26
2000–2200	0.28
2200–2400	0.28
2400–2600	0.27
2600–2800	0.28
2800–3000	0.32
3000–3200	0.27
3200–3400	0.31
3400–3600	0.28
3600–3800	0.27
3800–4000	0.28
4000–8000	0.28

We estimate two alternative within-herd transmission (Susceptible-Occult-Reactive-Infectious: SORI, Susceptible-Occult-Reactive: SOR) models that differ in terms of the assumed timing of the onset of infectiousness. The SORI model is the more traditional view of bTB progression in cattle [22–24] where susceptible animals (S) must progress through a series of latent classes where they are first undetectable (or occult O), detectable (or reactive R) before finally becoming infectious (I). The SOR model accounts for evidence for the potential ‘early’ transmission of bTB [25] and assumes all infected animals are potentially infectious, but still differ in their detectability.

Following [7], we use a Sequential Monte Carlo implementation of Approximate Bayesian Computation (ABC-SMC) [26] for estimation of our models. Within this methodology, inference of model parameters is based on a set of goodness-of-fit metrics, stratified here by herd size, parish testing interval (PTI) and whether bTB has been confirmed by culture (OTF-S or OTF-W status). Until recently, the frequency of routine testing for herds was determined by the historical herd-level incidence within a parish. As such, for our study population, the frequency at which herds were tested is also a proxy measure for the background risk of infection.

**Fig 2:**
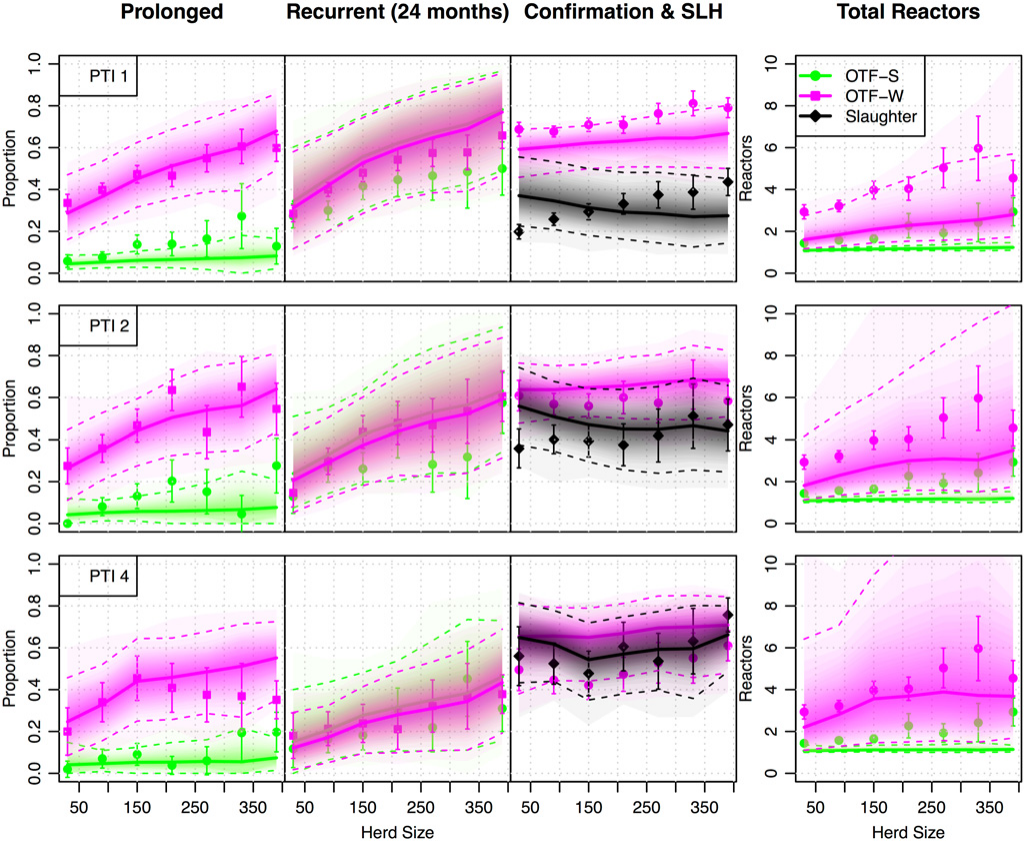
Persistence and surveillance metrics for bTB in GB herds (2003–2011). Within-herd measures of persistence and surveillance used as target metrics for ABC and to assess model fit. We present four key measures, from left to right: the proportion of prolonged (restrictions of greater duration than 240 days) and recurrent breakdowns, the proportion of herds with evidence of visible lesions and the total number of reactors per breakdown. Breakdowns are classified as either OTF-S (officially TB free suspended), where no reactors are found to have visible lesions (lime green circles), or OTF-W (officially TB free withdrawn) where at least one reactor was found to have evidence of visible lesions or be culture positive (magenta squares). The proportion of such OTF-W breakdowns is shown along with the proportion of these that were initiated by a slaughterhouse case (black circles). The relationship of each measure with herd size is plotted, with breakdowns further stratified by the historical parish testing interval (A, PTI1; B, PTI 2; C PTI 4) and breakdown status. Mean target observations are plotted with uncertainty estimated as ±1.96 standard errors around the mean. Predictive distributions from our within-herd (SORI) model for each of these measures are plotted as shaded density strips where the intensity of color is proportional to the probability density at that point [34].

We achieve a comparable fit to our previously published models with respect to the proportion of prolonged and recurrent breakdowns, both indirect measures of the within-herd persistence of infection (Fig. 2). In common with our earlier study [7], both models underestimate the proportion of OTF-W breakdowns in high incidence (PTI 1) areas and overestimate the same proportion in low incidence (PTI 4) areas. However, this discrepancy is far smaller than before with the majority of target values lying within the envelope of the 95% predictive intervals.

Likewise, both models still underestimate the mean number of reactors, in particular for breakdowns with no evidence of visible lesions (OTF-S) and for confirmed breakdowns (OTF-W) in PTI 1. Herd level incidence, as measured by the number of reactor animals removed during a breakdown, is remarkably consistent across testing intervals (Fig. 2). Although the predictive distributions are more variable, our new models still struggle to capture this pattern. These patterns arise as incidence in high-risk (PTI 1) herds is strongly constrained by the requirement to fit the incidence rate in low-risk PTI 4 herds where infectious animals can potentially remain within a herd for a longer period of time between routine testing. Classification of herds purely by testing interval is a crude approximation given that reservoirs of bTB in local wildlife is likely to generate considerably heterogeneity in the risk of background infection down to the herd level. Further heterogeneities not accountable within our modeling framework such as geographic variation in the efficiency of surveillance in slaughterhouses [27] or during routine testing [28] are also likely to contribute to this lack of fit.

**Fig 3:**
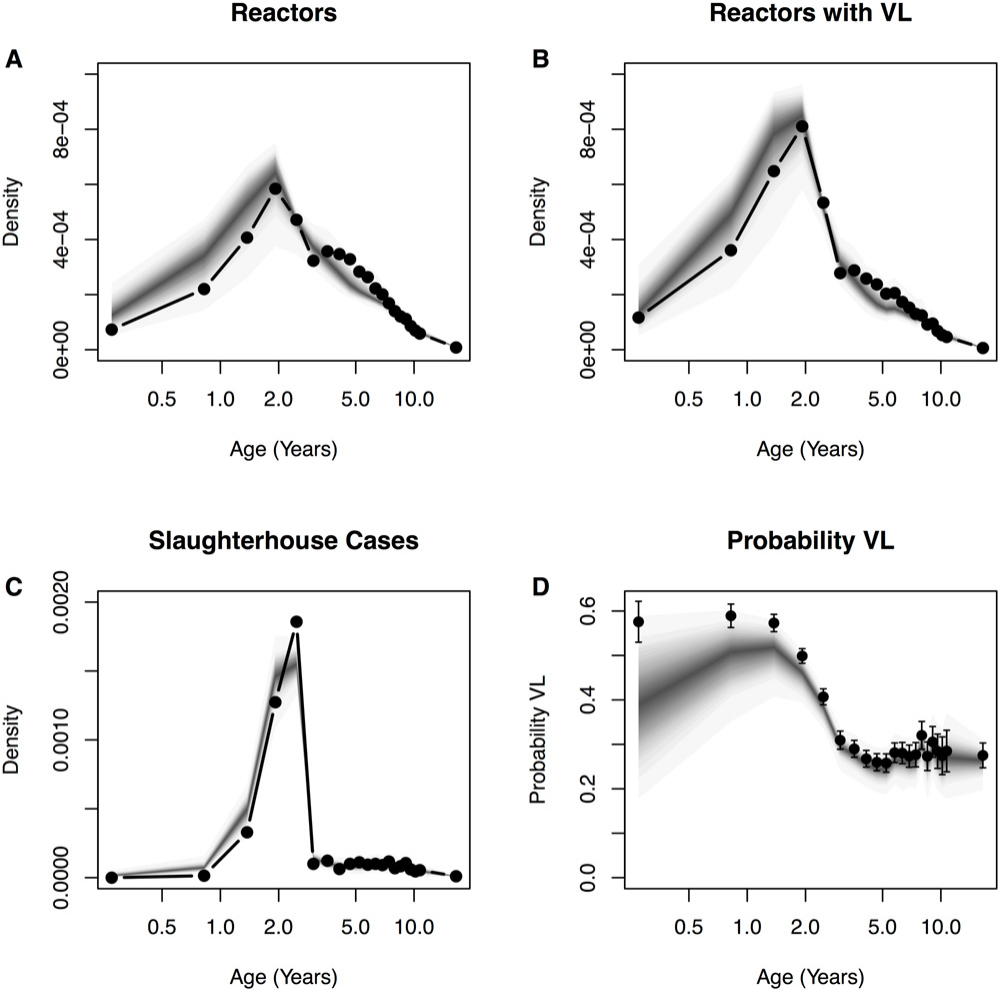
SORI model fit to age-distributions of reactors. SORI model predictive distributions for the age of reactors (**A**). Age of “confirmed” reactors with evidence of visible lesions (**B**). Slaughterhouse cases (**C**) and the proportion of animals with visible lesions stratified by age (**D**). Solid points and lines indicate empirical target distributions, model predictive distributions are once again overplotted as shaded density strips where the intensity of color is proportional to the probability density at that point [34].

The model captures the broad characteristics of the age distribution of reactor animals, slaughterhouse cases and visibly lesioned animals (Fig. 3). However, the assumption that animals are immediately replaced on removal from herds does introduce a small bias in the instantaneous age-distribution of herds with a consequently higher proportion of young (< 2 years) animals and reactors (Fig. 3A,B). There is also a notable discrepancy between the empirical estimates for the proportion of animals with confirmed *M*. *bovis* and model predictions (Fig. 3D). This is the consequence of approximating the age-dependent probability of confirmation of reactors using empirical estimates from reactor animals (Table 2). Laboratory confirmation of lesions is assumed to be 100% specific, so logically we must only simulate the confirmation process for reactors from infected compartments. As a consequence, false positive reactors reduce the proportion of reactors with visible lesions in simulated output, with the greatest discrepancy in younger reactors that are less likely to be infected.

Estimates of model parameters are more uncertain than in [7] as a consequence of improved mixing of parameters in the ABC-SMC procedure and a more realistic representation of inter-herd variability (S1 Fig.). Several parameters are poorly identified, in particular the reactive period, occult period, slaughterhouse detection parameter and density dependence parameter. This is a consequence of strong trade-offs, in particular between transmission and surveillance parameters. These trade-offs lead to, in the terminology of Gutenkunst et al. [29], ‘sloppy’ approximate posterior distributions where a large degree of variability has only a small impact on model predictions.

Once again we find no clear evidence to choose between the two alternative models. However, as we shall see, for the purposes of exploring strategies for vaccination it is DIVA test characteristics that are of primary importance, and interest. For simplicity we only present the SORI model results, but include equivalent simulations of the SOR model as supplementary information (S2–S6, S10 Figs.).

### Vaccination scenarios

Vaccination is modeled by tracking vaccinated animals, recording the epidemiological status of each (S7 Fig.). Vaccinates are assumed to have the same rates of disease progression as unvaccinated animals, but differ only in their reaction to diagnostic tests and in having a reduced rate of infection by a factor of ε = (1—individual animal vaccine efficacy). We consider a set of three vaccination scenarios using different combinations of tuberculin and DIVA testing motivated by discussions with UK government policy teams. All scenarios are based upon an annual re-vaccination of herds in concert with annual herd tests. The **DIVA Negate** scenario adheres most closely to the current regulatory regime where all animals are tuberculin tested, with tuberculin positive vaccinates subject to an additional DIVA test to attempt to negate false positive reactors. For the **DIVA Replacement** scenario tuberculin testing is only used for unvaccinated animals, with vaccinates tested only using a DIVA test. Finally, we consider a strategy where restrictions are lifted based on the number of animals with confirmed *M*. *bovis* lesions rather than DIVA positivity. Under the **VLend** scenario testing is carried out as under **DIVA Replacement**. However for VLend, short interval testing can be suspended after two clear tests or two successive tests where DIVA reactors are found to have no confirmed lesions.

We compare each of these scenarios, by challenging newly vaccinated herds with a single infected animal onto the herd. Benefits of vaccination will only be manifested if the vaccinal protection is sufficient to offset any increase in the number of animal tests, and false positive reactors, generated by either sensitization to tuberculin or the incomplete specificity of DIVA testing. We use four key measures of the epidemiological and economic costs associated with bTB and testing to assess the benefit of vaccination at the herd level: the number of animals condemned as reactors (R); the number of tests (tuberculin and DIVA) needed to clear restrictions (T); the number of infected animals left in herds after restrictions are lifted (burden of infection missed by testing, B) and finally the number of herds that experience a breakdown before the herd clears the singleton challenge (N).

### Vaccine efficacy and herd level benefit using γ-interferon DIVA test

**Fig 4:**
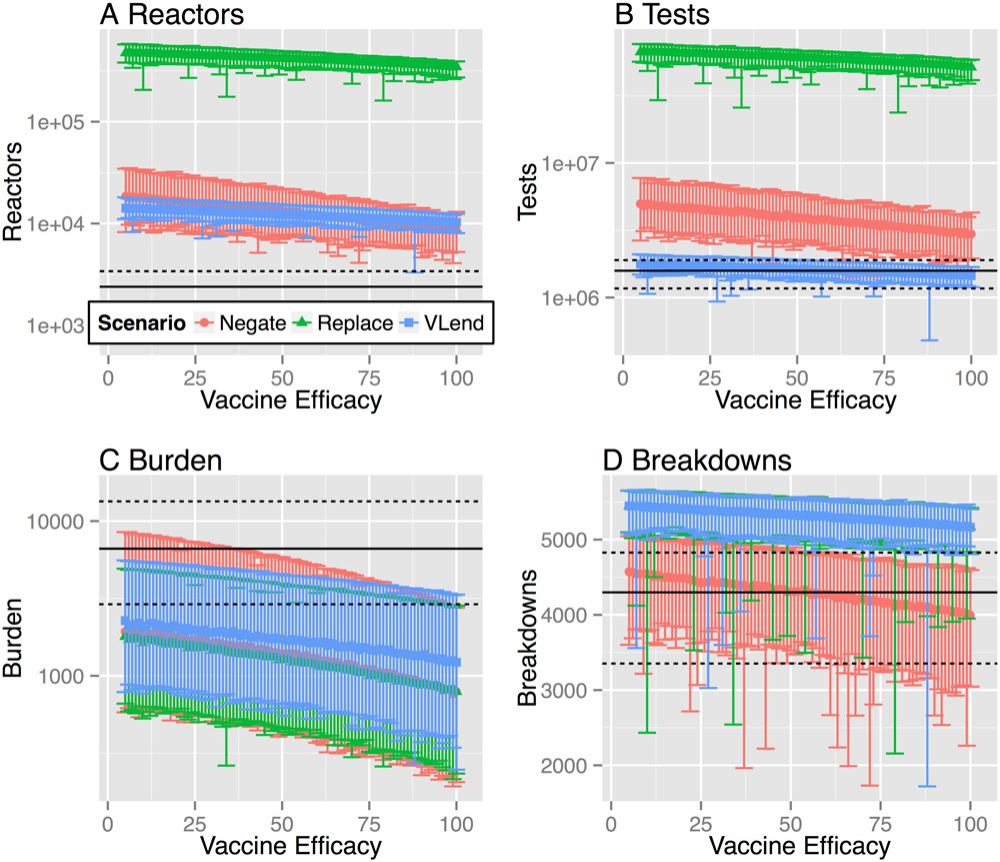
Break-even points for vaccine efficacy under alternative testing scenarios using γ-DIVA test. We estimate the break-even point for a protective benefit of BCG vaccination at the herd level under three alternative testing scenarios. We model DIVA testing using parameter estimates that optimize DIVA specificity of 99.4% under the constraint of maintaining a DIVA sensitivity comparable to tuberculin testing of 64.4%. We consider four key measures of the epidemiological, and economic, costs associated with bTB testing: **A** the number of animals condemned as reactors; **B** the number of tests (tuberculin and DIVA) needed to clear restrictions; **C** The number of infected animals left in herds after restrictions are lifted (burden of infection missed by testing) **D** The number of herds that experience a breakdown before the herd clears the singleton challenge. For all panels, solid black lines indicate the median break-even point for the baseline scenario with no vaccination. Dashed lines indicate the 95% quantiles of the baseline scenario. The distribution for each measure is calculated from 100 simulations with parameters drawn from the (approximate) posterior distributions of our estimated model, with each parameter set simulated once for each herd within our representative study population (of 6,601 herds).

Although proof-of-concept experiments have been carried out for a DIVA skin test [9] at the current time the only viable DIVA test for use in cattle is based on the γ-interferon blood test platform [8]. This test is similar to comparable tests used to diagnose human TB, such as the Quantiferon Gold or TSpot assays and with comparable or better performance characteristics [30]. In common with the SICCT, the interpretation of the γ-interferon DIVA test can be adjusted to trade off sensitivity and specificity of the test. The current candidate γ-interferon DIVA test depends on a combination of response to three antigens: ESAT-6, CFP-10 and Rv3615c. Data to characterise the response of these antigens have been collected from experimentally *M*. *bovis* infected animals (107 for ESAT-6/CFP-10, 109 for Rv3615c), non-infected controls (874 for ESAT-6/CFP-10 and 481 for Rv3615c) and a smaller data set of BCG vaccinated/experimentally infected animals (75) and BCG vaccinated non-infected controls (214). We initially selected cut-off values for the three antigens that maximize the DIVA specificity in the larger data set of unvaccinated animals. Applying these cut-off values to the smaller data set of BCG vaccinates provides an estimated DIVA sensitivity of 64.4% (95%CI: 48.8, 78.1%) and 99.4% specificity (95%CI: 96.9–100% CI). Based upon these DIVA test characteristics we simulated our estimated model over the range of individual protective efficacies *ε*~[0,1] (Fig. 4 and alternative version with linear scale S9 Fig.).

Across the scenarios considered, vaccination provides a clear protective benefit only in terms of the residual infection remaining on herds that clear movement restrictions (Fig. 4C). This marginal benefit comes at a considerable cost in terms of the number of tests necessary to clear restrictions on herds and the number of animals condemned as reactors under DIVA testing (Fig. 4A,B). Even if we assume that vaccination has 100% efficacy, we fail to see a herd level benefit of vaccination with over a three-fold increase in the number of animals condemned as reactors, with a median estimate of 8,663 reactors under the **DIVA negate** strategy (red) compared to 2,392 under the baseline, no vaccination scenario. Under this same comparison, **DIVA replace** is by far the most inefficient strategy with a 32-fold increase in the number of tests (median estimate of 51,554,010 compared to 1,582,048) and 144-fold increase in the number of reactors (median estimate of 345,775 compared to 2,392), despite the assumption of 100% efficacy. This difference is driven almost completely by a high rate of false positive reactors due to the lower DIVA specificity. These testing costs are mitigated through the **VLend** strategy, although the number of animals removed as reactors is still higher than in the absence of vaccination (median estimate of 10,277 compared to 2,392).

The relative impact of vaccination within these scenarios is driven almost completely by the specified test characteristics of the DIVA test, in particular the impact that the relatively low DIVA specificity compared to SICCT testing has when used within the intensive testing regime used for GB herds. As such, we expect these results to be largely insensitive to model choice for within-herd transmission, and indeed we see qualitatively comparable patterns for the alternative SOR model (see Supplementary Information). This simulation study demonstrates the key importance of DIVA test performance, rather than individual vaccine efficacy, in determining the cost-effective use of BCG vaccination in cattle. To explore this question in more detail we quantify the break-even points for seeing a protective herd level benefit of vaccination as a function of DIVA test characteristics.

### Break-even points for DIVA sensitivity and specificity

To quantify the expected break-even point for DIVA test characteristics we performed a broad parameter sweep of assumed values of DIVA sensitivity and specificity under our three alternative testing scenarios. For this comparison we fix efficacy at ε = (1–0.61), corresponding to an individual level efficacy of 61%. We focus on three of our proxy measures relating to costs of testing: the number of breakdowns (N), number of tests required to clear restrictions (T) and burden of infection remaining (B). We calculate the probability of seeing a protective benefit by comparing the distribution of each proxy measure to the baseline distribution for tuberculin testing alone. These distributions are approximated by 100 posterior draws from our estimated model and under the vaccination scenario. The probability of benefit is calculated as the proportion of independent samples where the value from the vaccination scenario is less than the sample from the baseline scenario.

This probabilistic measure captures the uncertainty and variability in both the parameter estimates of our model and due to demographic stochasticity. We consider the break-even point to be when the probability (proportion) of benefit relative to the baseline of tuberculin testing only is 0.5.

**Fig 5:**
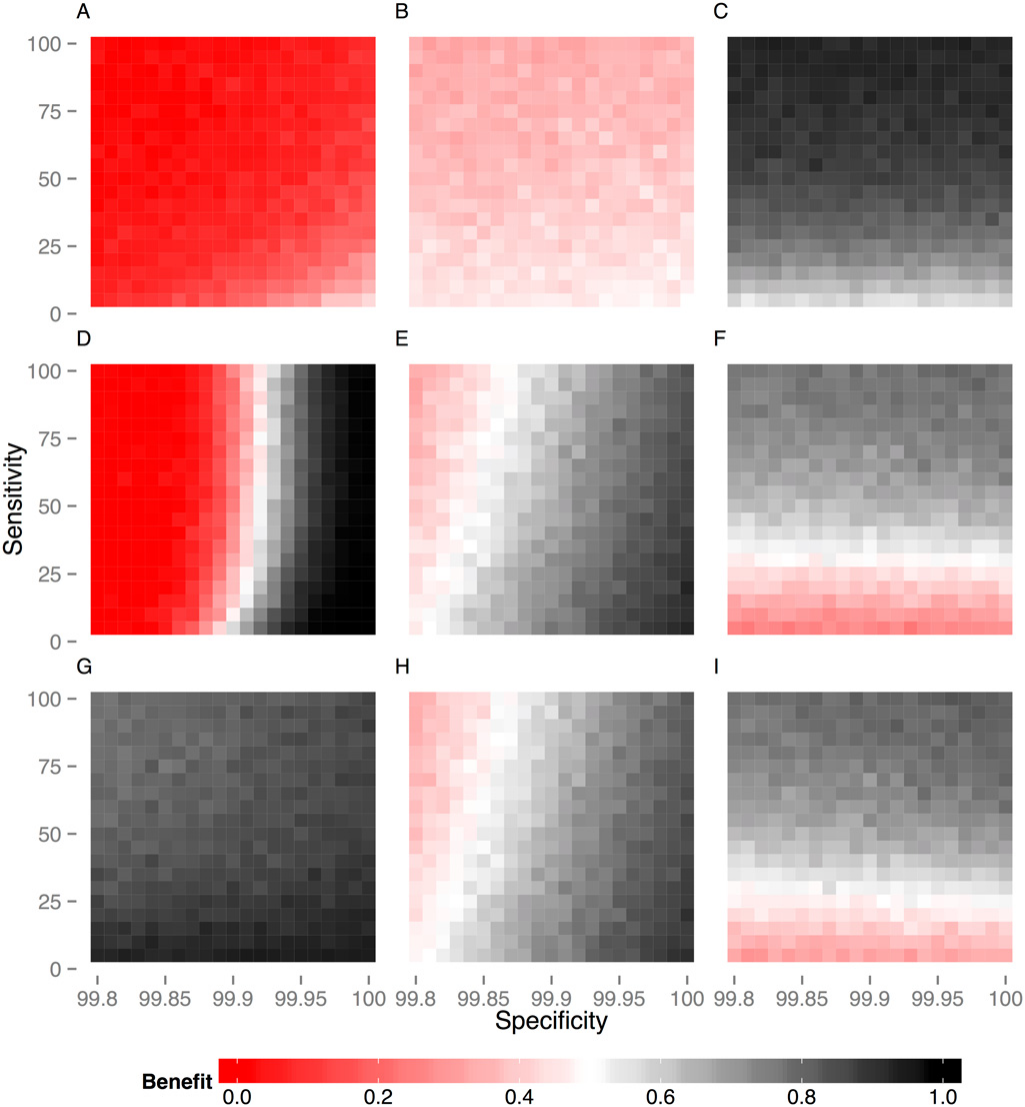
Probability of a protective herd level benefit of vaccination for alternative DIVA testing strategies. We explore how the probability of seeing a protective benefit of vaccination depends on the assumed sensitivity and specificity of DIVA testing. The probability of benefit is calculated for a singleton challenge of infection and relative to the current statutory regime of tuberculin testing and slaughterhouse surveillance. Benefit is estimated from 100 simulations of our study population (6,601 herds) for three key measures (across columns): the total number of tests required to clear restrictions (**A,D,G**), the probability of restrictions being applied before the herd clears infection (**B,E,H**) and the probability of infection remaining in a herd when restrictions are lifted (**C,F,I**). We define the break-even point as 50% of herds demonstrating a protective benefit illustrated by the white band in the color map with red values worse this threshold and grey points better. We compare the three strategies described in the main text (across rows): (**A,B,C**) Under the DIVA negation scenario, the break-even point is limited by the considerable overhead in testing, with an increased probability of restrictions being applied before the herd clears infection (**B**) and an increase in testing (**A**) even for a 100% sensitive and specific DIVA test. (**D,E,F**) Under DIVA replacement, a protective benefit of vaccination can be achieved for DIVA specificities > 99.90% (**D**). The break-even point also depends on DIVA sensitivity, with a sensitivity of at least 40% being necessary to avoid increased risk of leaving infection in the herd after restrictions are lifted (**F**). (**G,H,I**) Under the VLend scenario, linking the maintenance of restrictions to detection of lesioned reactor animals mitigates the addition costs of testing under other scenarios (**G**). However, a specificity of greater than 99.85% is still required to see no increase in the number of breakdowns with vaccination (**H**), with the break-even point depending again on a DIVA sensitivity of greater than 40% (**I**).

**DIVA negation**. Although DIVA negation provides a benefit when DIVA specificity takes the estimated values used above (Fig. 4) the **DIVA replacement** and **VLend** scenarios are clearly more efficient over a wider parameter range than **DIVA negation** provided the DIVA specificity can be raised to a sufficiently high value (Fig. 5). Under **DIVA negation** the additional tests needed to negate tuberculin positive vaccinates overwhelms any protective benefit of vaccination in terms of the number of tests (Fig. 5A) and probability of breakdown (Fig. 5B), even for a DIVA specificity of 100%. It should however be noted that in terms of the number of tests carried out the maximal probability of benefit under DIVA negation occurs when the sensitivity of testing is zero and no animals are removed under testing. In this extreme scenario the protection afforded by vaccination is sufficient to reduce the burden of infection to a comparable level as tuberculin testing (Fig. 5C) with the increase in duration (and frequency) of breakdowns driven entirely by false positive tuberculin reactions.

**DIVA replacement**. Under **DIVA replacement**, a DIVA specificity of above 99.90% in combination with an absolute sensitivity of at least 40% can provide a protective benefit of vaccination relative to tuberculin testing alone (Fig. 5 D,E,F). This required DIVA specificity is comparable to the lower bound we place on the specificity of the severe interpretation of the SICCT test for our model estimates. Once again it is important to note that the relationship between the probability of benefit and expected costs with DIVA sensitivity is non-linear, with highly sensitive tests potentially increasing the costs associated with testing at the herd level (Fig. 5D).

**VLend: Changing the endpoint of breakdowns**. Changing the endpoint of breakdowns to depend on evidence of visible lesions in reactor animals can demonstrate a protective benefit across all three cost measures (Fig. 5 G,H,I) for comparably lower values of DIVA specificity (although still very high at >99.85%) and sensitivity than under **DIVA negation** or **DIVA replacement**. Although the negative impacts of DIVA specificity are mitigated under this scenario, as a consequence DIVA sensitivity is more important; a break-even sensitivity of ~40% is constrained by the risk of leaving infection within the herd (Fig. 5I). Finally, it should be noted that the magnitude of the benefit under the **VLend** scenario compared to tuberculin testing would be considerably less if we used the same end-point definition (i.e. in terms of animals with VLs) for tuberculin testing in the absence of vaccination.

## Discussion

There has recently been a shift in the political landscape with respect to the position of the EU on introduction of BCG vaccination for cattle. The requirements for changes in legislation to allow the use of BCG by member states have been detailed in a recent EFSA opinion [19]. Of central importance is the demonstration of the performance of a suitable DIVA test in large-scale field trials under European production conditions. The potential risks, and outstanding challenges, associated with achieving this goal are highlighted in this study.

For vaccination to be tenable it must not only be effective epidemiologically, but must be cost-effective for individual farmers as well. When the interpretation of the interferon-γ DIVA test is calibrated to have a comparable sensitivity to SICCT testing, the low specificity relative to SICCT testing is predicted to considerably increase the costs associated with testing for all three scenarios we considered. Under this interpretation and for an individual level efficacy of 61%, the median number of tests required to clear restrictions for our study population increases from a baseline estimate of 1,582,048 in the absence of vaccination to 3,674,010 for **DIVA negation**, 58,973,457 for **DIVA replacement**, but remains comparable at 1,592,710 for the **VLend** scenario. In model simulations we constrain herds to have a constant size. In practice the level of false positives generated by the interferon-γ DIVA test as modeled here could lead to the depopulation of whole herds unless the regulations governing the sequencing of breakdown testing are relaxed (as suggested by the **VLend** scenario).

The recent EFSA opinion [19] focuses on the requirement that any prospective DIVA test must be of comparable efficacy to tuberculin testing. This comparison raises considerable questions as the efficacy of tuberculin testing itself depends on the interpretation of the test and the relationship between test-positivity and visible lesions. The mechanisms underlying the age-dependence of reactor rates and lesions with age (Fig. 1) are still poorly understood, as is the impact that vaccination may have on this picture.

Our herd level models provide an objective measure of the efficiency of testing through the burden of infection left on herds when restrictions are lifted. Our models suggest that this burden of infection can be reduced in vaccinated herds even when DIVA sensitivity is lower than SICCT testing—provided that the individual level protection is great enough. Analyzing a recently obtained larger data set (Jones, Vordermeier, personal communication) from BCG vaccinated and BCG vaccinated/experimentally *M*. *bovis* infected cattle we explored the potential to reach this target DIVA specificity in vaccinated animals. We estimate that a relative sensitivity of the DIVA test of 73.3% (95%CI: 61.9, 82.9%) (using ESAT-6, CFP-10, Rv3615c antigens) can be achieved at the break-even specificity level of > 99.85. This is comparable to the lower end of estimates of tuberculin testing in the literature [11]. The potential misclassification of infected individuals by this imperfect gold standard measurement complicates the translation of empirical estimates of test characteristics to model parameters. In the absence of a true gold-standard test, the relationship between absolute test characteristics and relative measures cannot be modeled *a priori*. Care must therefore be taken in comparing between the absolute test sensitivity and specificities used as model parameters and estimates relative to the presence of visible lesions (which is as previously discussed an imperfect diagnostic test itself).

Our analysis demonstrates the challenges inherent in bTB control where we have no gold standard diagnostic test or clinical manifestation of disease. Incidence and targets for control of bTB are based on the estimates of prevalence inferred from tuberculin testing. As a consequence, even if an improved diagnostic test was available it could, at least in the short term, paradoxically apparently increase incidence and the economic costs associated with control. The **DIVA negate** and **DIVA replacement** scenarios considered in this report demonstrate that reducing the sensitivity of testing can offset negative effects of imperfect specificity. The corresponding risk of increasing the probability that infection is left within herd can be estimated from models, however monitoring and quantifying this risk would be an essential requirement for any prospective field trial. In all of the scenarios we have considered vaccination reduces the burden of infection within herds, and thus the risk of herds moving infected animals when restrictions are lifted.

Under the current test-and-slaughter regime, the outcome of a bTB breakdown, in practice and as captured by our models, is highly variable. Systematic heterogeneities between herds and individuals contribute to this variation, as does uncertainty in the life history of infection. Fundamentally, this variability is driven by the intrinsically stochastic dynamics of transmission, detection, and removal of infected animals. For each of our models we aim to capture the extent of this variability through estimating distributions of parameters rather than point estimates. As a consequence the predicted outcomes of vaccination from our models are highly variable (Fig. 4). We therefore chose to use probabilistic measures define the break-even point for vaccination scenarios at the population level (Fig. 5). It is important to acknowledge that the intrinsic stochasticity of bTB transmission limits the predictive ability of models and means that strategies that provide an overall benefit at the population level will not guarantee positive benefits for individual farms.

In conclusion, we found that the efficacy of BCG is largely irrelevant with respect to seeing a benefit when BCG is introduced with respect to the frequency and duration of restrictions applied to farms. DIVA specificity, rather than sensitivity, is the biggest barrier to the efficient use vaccination and indeed to the economic feasibility of field trials of BCG vaccination. Derogations with respect to the requirement of tuberculin testing vaccinated animals, or changing the sequence of testing that herds must pass to clear restrictions may help to mitigate these negative effects. In order to break even under even the most liberal changes to testing considered in this report (**VLend**) would require a DIVA specificity of > 99.85%. This value provides a clear requirement for validation of prospective DIVA tests before deployment in the field.

## Methods

### Study population

Our within-herd modeling framework estimates rates of transmission and removal of infection from herds based on the strict timescales imposed by the regulatory structure of the testing regime in Great Britain. The sequence of tests following disclosure of reactor animals places strict bounds on the duration of time that infection can remain undisclosed within herds. This is not necessarily true for breakdowns initiated by tracing tests, contiguous testing, inconclusive reactors or follow-up tests following a breakdown. To control for this extra source of variation in the time-to-detection we restrict our study population to “new” breakdowns that were disclosed by routine surveillance tests (classified as VE-WHT, VE-WHT2,VE-RHT,VE-SLH). To limit the impact of disruptions to testing during the Foot and Mouth Epidemic of 2001 and to allow enough follow up time to estimate the probability of breakdowns recurring we further limit our study population to breakdowns with start dates after 1/1/2002 and end dates before 1/9/2009. In a departure from our previous study we place no upper bound on the size of herds, but do require that herds had a minimum size of 10 animals on the start date of the breakdown and at least 30 CTS records for the associated CPH. Finally, we excluded any breakdowns where there was discretionary use of γ-interferon testing. This provides us with a study population of 6,601 herds containing 1,170,541 cattle on the start date of their breakdown.

### Target metrics for ABC

We estimate the parameters of our within-herd models using an Approximate Bayesian Computation (ABC) method as described in [7]. ABC is a systematic framework for parameter inference when the likelihood function is intractable or computationally expensive [31]. Parameter estimation is performed through approximating a likelihood function through the probability of matching model simulations to the observed data using a set of goodness-of-fit metrics. For our within-herd models, these metrics consist of a set of within-herd persistence measures related to the duration and probability of recurrence of breakdowns stratified by the historical parish testing interval and herd size (Table 1).

For the purpose of model checking we consider an additional set of age-stratified metrics: the age-distribution of reactor animals, the age-distribution of reactor animals with evidence of visible lesions, the age-distribution of slaughterhouse cases and the probability of visible lesions with respect to age (Fig. 1B, Table 2). For the purpose of reproducibility, all target metrics and anonymised data for each of the study herds are provided as supplementary data tables (S1 Dataset).

### ABC-SMC methodology

As in [7] we use the ABC-SMC algorithm of [26]. We sampled a set of 1,500 sets of parameters or particles (Table 3), from a set of uniform prior distributions (Table 4). For each set of parameters we simulate the model for a single replicate for each of the 6,601 herds in our study population and calculate the discrepancy between simulations and our set of target metrics (Table 5). This is a departure from [7], where we simulated multiple (500) replicates of the model at the median value of the herd-size used to stratify our persistence metrics. The new method generates more variable simulated output that is more representative of the variability intrinsic to the size and characteristics of the data set. We then simulate successive rounds where we accept and reject proposed particles according to a set of tolerances set on our summary metrics (described above). Newly proposed particles are sampled from the previous round and perturbed using a multivariate normal kernel with twice the sample variance estimated from the previous round. Convergence of the method is assessed through comparison of the predictive distributions simulated from the model to our set of target metrics (Figs. 2, 3) after 30 rounds of ABC-SMC, reducing the tolerance at each step to the 90th percentile of the particles in the previous round.

**Table 3:** Model parameters.

Parameter	Description
p_T_	**Standard SICCT Sensitivity**. Probability of positive tuberculin test for (R,I) individuals at standard definition.
1-*p*_*FP*_	**Standard SICCT Specificity**. Probability of negative tuberculin test for S,O,R,I individuals at standard definition. (1—probability of a false positive *p*_*FP*_)
${p^{\prime }}_{T}$	**Severe SICCT Sensitivity**. Probability of positive tuberculin test for R,I individuals at severe definition
$1-{p^{\prime }}_{FP}$	**Severe SICCT Specificity**. Probability of negative tuberculin test for S,O,R,I individuals at severe definition.(1—probability of false positive${p^{\prime }}_{FP}$)
p_RI_	**Slaughterhouse detection**. Relative sensitivity of finding lesioned or culture positive animals (R,I status) under routine inspection compared to reactor inspection
T_0_	**Occult Period**. Mean length of time that animals are undetectable (occult) to SICCT
T_R_	**Reactive Period**. Mean length of time between infection and animals becoming infectious
β	**Transmission parameter** associated with density dependence (rate per day, dimensions change with q)
q	**Transmission parameter** measuring the strength of density dependence (range 0–1)
*χ*_1_	**Transmission parameter** measuring infectious pressure per susceptible per year in PTI 1
*χ*_2_	**Transmission parameter** measuring infectious pressure per susceptible per year in PTI 2
*χ*_4_	**Transmission parameter** measuring infectious pressure per susceptible per year in PTI 4
*H*_*m*_ = 165	**Constant** equal to mid-point of range of herd sizes within study population. Used to transform density dependence of force of infection.

**Table 4:** Prior distributions.

Parameter	Prior Constraints	Initial sampling distribution
p_T_	0.01<*p*_*T*_<1	Uniform [0.01, 1]
1-*p*_*FP*_	0<*p*_*FP*_<0.0003	Uniform [0.0,1–0.9997]
${p^{\prime }}_{T}$	$p_{T}<{p^{\prime }}_{T}<1$	Uniform [*p*_*T*_,1]
$1-{p^{\prime }}_{FP}$	$0.001>{p^{\prime }}_{FP}>p_{FP}$	Uniform [0,1–0.9990]
P_RI_	0<*P*_*RI*_<1	Uniform [0.0,1.0]
*T*_0_ (SOR)	0≤*T*_0_≤0.35	Uniform [0.0,0.35]
*T*_0_ (SORI)	0≤*T*_0_≤0.35	Uniform [0.0,0.35]
*T*_R_	0≤*T*_0_≤10	Uniform [0.0,10.0]
β	0≤*β*≤0.1	Uniform [0,0.1]
q	*0<q*<1	Uniform [0,1.0]
*χ*_1_	0<*χ*_1_<10/(1000×364)	Uniform [0,10/(1000*364)]
*χ*_2_	0<*χ*_2_<10/(1000×364)	Uniform [0, 10/(1000*364)]
*χ*_4_	0<*χ*_4_<10/(1000×364)	Uniform [0, 10/(1000*364)]

**Table 5:** Epidemiological target measures for ABC.

Description	Type of Measure	Number of bins per target distribution	**Weighting (${w^{\prime }}_{j}$**)
Breakdown Length	Distribution (Days) [100,200,300,400,500,1000,2000]	7	1/7
Total reactors removed within breakdown (until movement restrictions are lifted)	Distribution (Reactors) [2,4,6,8,10,12,14,16,18,20,47]	11	1/11
Proportion of breakdowns recurring within 24 months	Probability	1	1
Proportion of breakdowns confirmed	Probability	1	1
Proportion of confirmed breakdowns started by slaughterhouse case	Probability	1	1

### Simulation algorithm

Our epidemiological models are implemented in an individual based framework where we track information on each animal within the population (Table 6), rather than the number of individuals within given epidemiological compartments (S,O,R,I…). Epidemiological events are modeled as a continuous-time Markov process [32] defined by the list of events and rates for the SORI and SOR models detailed in Tables 7 and 8. The demographic events of birth, death and movement off a herd are simulated as a non-Markov process. The time of each of demographic events is sampled from an extract of the CTS database for each individual animal added to the model. During each step of the model we simulate the time for the next Markov event (t_MARKOV_) and compare to the time of the next non-Markov (demographic) events (t_NON MARKOV_). If t_NON MARKOV_ < t_MARKOV,_ we carry out the demographic event first and recalculate a new t_MARKOV._ Otherwise a Markov (epidemiological) event is simulated and the model time updated.

**Table 6:** Individual animal variables.

Data Field	Data Type	Description and Notes
Epi_status	Enumeration (S,O,R,I,V1,V2,OV1,OV2,RV,IV)	Epidemiological status
Birth_time	Numeric (Days)	Used to track age of individuals
Death_time	Numeric (Days)	Used to schedule removal from herd and book-keep denominator for slaughterhouse surveillance
Off_time	Numeric (Days)	Used to schedule removal from herd and book-keep denominator for slaughterhouse surveillance
Vaccinated_time	Numeric (Days)	Use to calculate probability of being SICCT positive

**Table 7:** Markov events for SORI(V) stochastic transmission model.

Event	Status	Effect	Probability per unit time
Infection	S	*S*→*O*	$\left(\frac{\beta I+\chi }{{\left(H/H_{m}\right)}^{q}}\right)\times RR(a)$
	*V*_2_	*V*_2_→*O*_*V2*_	
Infection	*V*_1_	*V*_1_→*O*_*V1*_	$\varepsilon \left(\frac{\beta I+\chi }{{\left(H/H_{m}\right)}^{q}}\right)\times RR(a)$
Emergence (Occult)	0	*0*→*R*	(1/*T*_0_)
	O_V1_	*O*_*V*1_→*R*_*V*_	
	O_V2_	*O*_*V*2_→*R*_*V*_	
Emergence (Reactive)	R	*R*→*I*	(1/*T*_*R*_)
	R_V_	*R*_*V*_→*I*_*V*_	
Loss of Protection	*V*_1_	*V*_1_→*V*_2_	(1/*T*_*V*_)

**Table 8:** Markov events for SOR(V) stochastic transmission model.

Event	Status	Effect	Probability per unit time
Infection	S	*S*→*O*	$\left(\frac{\beta I+\chi }{{\left(H/H_{m}\right)}^{q}}\right)\times RR(a)$
	*V*_2_	*V*_2_→*O*_*V2*_	
Infection	*V*_1_	*V*_1_→*O*_*V1*_	$\varepsilon \left(\frac{\beta I+\chi }{{\left(H/H_{m}\right)}^{q}}\right)\times RR(a)$
Emergence (Occult)	0	*0*→*R*	(1/*T*_0_)
	O_V1_	*O*_*V*1_→*R*_*V*_	
	O_V2_	*O*_*V*2_→*R*_*V*_	
Loss of Protection	*V*_1_	*V*_1_→*V*_2_	(1/*T*_*V*_)

### Demographic events

When the herd is initialized, the birth, death and off-movement times for each individual animal are sampled using an extract from the CTS database (Table 9). This extract contains four pieces of information for each animal alive on the start-date of the corresponding breakdown for that herd within our study population: **HerdId** links the animal record to a unique herd within our study population: **OccupancyTime** is the number of days the animal remained on the herd after its first on-movement; **AgeOn** is the age of the animal when it was moved onto the herd (0 for births); **LifeTime** is the total number of days the animal lived (on any herd) and **BreakAge** is the age of the animal on the disclosing test of the breakdown associated with that herd in the study population. This information allows us to sample the timing of all of the demographic events occurring to an individual over its lifetime: time of birth, time of movement off herd, time of death.

**Table 9:** Individual animal demographic data extract from cattle Tracing System (CTSTable).

Herd Id	Occupancy Time	Age On	Life Time	Break Age
Unique herd ID	Days between animals recorded on-movement and off-movement from herd	Age of animal when moved onto herd (Days)	Days between animals birth and death records	Age of animal on start date of breakdown

For each animal added to a herd at the start of a simulation we sample a row (i.e. a set of values of **OccupancyTime**, **AgeOn**, **LifeTime** and **BreakAge**) from Table 9 and calculate the birth, death and off times for that animal:

Birth_time = (t-**BreakAge**) + unif(0,0.001)

Death_time = **Birth_time** + **LifeTime** + unif(0,0.001)

Off_time = **Birth_time** + **OccupancyTime** + unif(0,0.001)

Where t is the current time and unif(0,0.001) is a uniformly distributed random number in the range [0,0.001] added to ensure that each individual has a unique age, death and off movement time.

Herd size is initialized to the number of animals present on the herd at the beginning of breakdown associated with that herd. A constant population size is maintained by immediately replacing animals that are removed from the herd (through either demographic processes or testing) with a new animal. The birth, death and off times for each replacement animal are calculated in a similar way by sampling a row from Table 9:

Birth_time = (t-**AgeOn**) + unif(0,0.001)

Death_time = **Birth_time** + **LifeTime** + unif(0,0.001)

Off_time = **Birth_time** + **OccupancyTime** + unif(0,0.001)

In this way we model both births and cattle movements onto the herd, with the relative proportion of replacement animals through births and on-movements determined by the CTS extract for that herd and the value of **AgeOn**. The CTS extract for all herds in the study population is included as a supplementary data table (S1 Dataset).

### SICCT testing model

SICCT testing, and the sequence of tests before, during and after a TB breakdown is modeled as described previously in [7]. The sequence of tests before, during and after a breakdown is simulated by a model where the timing of tests and number of animals to be tested changes dynamically according to the state-variables of the epidemic model and the outcome of individual animal tests.

The model is initialised and then simulated forward, piecewise, between the dynamically scheduled tests before, during and for 5 years following the end of the first breakdown, or until a recurrent breakdown is triggered. The sequence of decisions following the outcome of herd tests is summarized in S8 Fig.

Simulations begin with the herd undergoing routine surveillance through slaughterhouse inspection and whole herd tests (classified as RHT or WHT) at 1, 2 or 4 yearly intervals corresponding to the historical parish testing interval (PTI) for that herd. Detection of a reactor animal triggers a breakdown. The herd then enters a sequence of short interval tests (SIT). Unconfirmed breakdowns end after a single clear test at the standard interpretation, while confirmed breakdowns must clear two tests—one at severe interpretation and the second at standard interpretation. Two follow-up tests, one six months after the end of a breakdown (VE-6M) and one 12 months later (VE-12M) are then scheduled. The time between all tests associated with a breakdown (SIT, VE-6M, VE-12M) are sampled from empirical distributions (S1 Dataset). The duration of time between routine tests is also sampled from an empirical distribution (with separate distributions for PTI 1, 2 and 4) to account for the additional variation in the time to detection that is a consequence of delays in testing and the transition of herds between different parish testing intervals (S1 Dataset).

For tests associated directly with a breakdown (SIT, VE-6M, VE-12M) the whole herd is tested. However, there is more variation in the type of test, and numbers of animals tested, in PTI 2 and 4 herds. This variability in the number of animals tested is the consequence of different eligibility criteria for animals based on their age and use. In our original models we described this variability with empirical distributions. Within the individual based model we can incorporate these eligibility criteria explicitly using the age of animals on the date of a test and their scheduled exit date from the herd (which is pre-calculated as described above). For whole herd tests (WHT) all animals older than 6 weeks are eligible for testing, whereas for routine herd tests (RHT) animals must be older than 2 years to be eligible.

Breakdowns are triggered by the detection of a reactor, either due to the presence of infected animals in the herd or the generation of a false positive test result. Nominally, we simulate the full sequence of tests until either of these events occurs with the proportion of false-positive breakdowns determined by the relative values of the specificity and the infectious pressure. In practice, and to increase the speed of simulations, this can be pre-calculated by explicitly calculating the probability of a false positive breakdown occurring between periods where there are no infectious animals within the herd.

### Age-stratified model of confirmation and slaughterhouse surveillance

As before, we simulate switching to the severe interpretation of the SICCT test used in OTF-W breakdowns after the detection of visible lesions (VLs) within reactor animals. However, our earlier models assumed that the probability of VLs being detected was constant per reactor—clearly inconsistent with the pattern of age-dependence in reactor rates and presentation of lesions (Fig. 1). Our current model uses this empirical pattern to simulate the number of lesioned animals in a test through a look-up table based on the animal’s age (Table 2). The lesion status of each individual reactor is then simulated as a Bernoulli trial.

Slaughterhouse surveillance also depends on the detection of visible lesions, or culture confirmation carried out as part of routine carcass inspection. As such, Fig. 1 implies that the efficiency of slaughterhouse surveillance should also depend on the age of the animal. We therefore model the efficiency of slaughterhouse surveillance as the conditional probability of detecting infected animals given that they have signs of visible lesions*P*_*SL*_(ɑ). We *define* the relative efficiency of slaughterhouse surveillance *P*_*RI*_ compared to inspection of reactor animals such that:

$$P_{SL}(a)=P_{RI}P_{VL}(a)$$

### Vaccination and DIVA testing, assumptions and model parameters

Modelling the impact of vaccination requires additional model parameters that describe the individual level impact of vaccination and test characteristics of vaccinated animals (Table 10). For all vaccination challenge scenarios, the herd is initialized with a whole herd vaccination and a single infectious individual. The herd is then re-vaccinated on a strict annual cycle after any scheduled whole herd test.

**Table 10:** Vaccination model parameters.

Parameter	Description
p_D_	**DIVA sensitivity** Probability of positive DIVA test in infected vaccinates (V_1_,V_2_,O_V1_,0_V2_,R_V_,I_V_).
1-*p*_*DFP*_	**DIVA specificity** Probability of negative DIVA test for uninfected vaccinates (V_1_,V_2_) (1—probability of a false positive *p*_*DFP*_)
ε	**Vaccine Efficacy** Reduction in risk of infection for vaccinates (status V_1_)
T_V_	**Protective Period**. Mean length of time that animals are protected by BCG vaccination (status V_1_)

Our understanding of the mode of action of BCG through which animals are protected is still incomplete. Our best estimates of vaccine efficacy come from natural transmission studies [4], [5]. In these studies vaccinated animals have been found to be free of disease suggesting that sterilizing immunity is possible. However, given the necessarily limited timescale of these trials it is also possible that BCG is simply acting to slow the rate of progression to detectable disease. Challenge experiments have demonstrated such an effect of BCG with a significant reduction in pathology in inoculated vaccinates, but no sterilizing immunity [2]. However, given that the effective dose under natural transmission may be lower than under artificial challenge these data do not rule out the possibility that BCG also reduces the susceptibility of individual cattle. Given that there are no data to inform the magnitude of the impact that BCG may have on rates of progression we choose to model the individual level protective effect of vaccination through a single parameter (ε) that describes the per-capita reduction in the risk of vaccinated animals acquiring infection. This choice of parameterisation also has the advantage of corresponding closely to the definition of vaccine efficacy as estimated from natural transmission studies.

Given the relative paucity of evidence described for reduction in the severity of disease for vaccinates, we assumed that rates of progression for infected vaccinates were unchanged. Loss of protection of vaccinates is modeled by introducing two vaccinate statuses (V_1_,O_V1_,V_2_,O_V2_) that are assumed to have the same basic test characteristics but differ only in their relative risk of infection (S7 Fig., Tables 7 and 8). Protection is assumed to be lost at a constant rate with the average duration of protection fixed at 1 year [2], [33]. Data on the effect of re-vaccination are lacking. In the absence of this important information we treat re-vaccinated animals in exactly the same way as newly vaccinated animals. As a consequence, our model may overestimate both the individual level protection of re-vaccinated animals and their sensitivity to tuberculin testing (as described below).

DIVA testing is modeled by an assumed DIVA sensitivity *p*_*D*_and specificity 1-*p*_*DFP*_ defined relative to the true infection and vaccination status of animals (Table 10). An important data gap should be highlighted, in that as with all bTB tests, the empirical estimates of current generation DIVA tests are estimated relative to the presence or absence of visible lesions. The potential misclassification of infected individuals by this imperfect gold standard measurement complicates the translation of empirical estimates of test characteristics to model parameters. In the absence of a true gold-standard test, the relationship between true test characteristics and relative measures cannot be modeled *a priori* and care must be taken in comparing between the absolute values of these parameters used within the model and relative values that would be estimated relative to visible lesions or culture.

### Tuberculin testing of vaccinates

Although the sensitizing effect of BCG vaccination on tuberculin testing is well known, quantitative data on the impact of BCG on test status are limited to a single challenge study carried out by AHVLA [6]. BCG vaccinates were SICCT tested at 90, 180, 270, 450 and 720 days post vaccination. Skin test measurements were then interpreted at the standard and severe interpretation and the probability of animals being classified as a reactor calculated (Table 11). We use these empirical data within our model to define the specificity of SICCT vaccinated animals. As a consequence of the sampling frequency of this experiment our model will underestimate the reactivity within the first 90 days post-inoculation (which we define as 0). There is a further data gap with respect to the sensitization after 2 years and on re-vaccination. We assume that the specificity of vaccinates takes a constant value post 720 days, and that re-vaccination leads to the same time-dependent pattern of sensitization relative to the time of revaccination. As a consequence of these assumptions our model will likely underestimate the number of false positive reactor animals in the first 90 days post-vaccination.

**Table 11:** Time-dependent sensitisation of vaccinates to SICCT [6].

Time from Vaccination (Days)	SICCT Interpretation	Probability classified as reactor
0–90	Standard	0.0
	Severe	0.0
90–180	Standard	0.60
	Severe	0.84
180–270	Standard	0.80
	Severe	0.95
270–360	Standard	0.09
	Severe	0.30
360–450	Standard	0.05
	Severe	0.10
450–720	Standard	0.11
	Severe	0.30
720+	Standard	0.10
	Severe	0.30

We model the sensitization of BCG vaccinates to tuberculin using a time-dependent probability of an animal being classified as a reactor estimated from challenge studies carried out by AHVLA [6]. The probability of being classified as a reactor is assumed to be constant with coarse-grained 90-day intervals and take the same constant value for all animals older than 720 days.

## Supporting Information

S1 Fig Parameter distributions estimated by ABC for SORI model. Parameter distributions estimated for SORI model by ABC-SMC. Distributions of parameters most consistent with persistence, surveillance and reactor distributions estimated from VetNet data (Summarised in Fig. 1) conditional on our prior assumptions (Table 4). Each approximate posterior distribution is plotted on the range of the (uniform) prior distributions for each parameter. Sensitivity and specificity parameters are further constrained such that the severe interpretation always has a higher sensitivity and lower specificity (Panels A,B). On panel B a dashed vertical line indicates the lower bound of the prior distribution for specificity at the standard interpretation.
(TIF)

S2 Fig Parameter distributions estimated by ABC for SOR model. Parameter distributions estimated for SOR model by ABC-SMC. Distributions of parameters most consistent with persistence, surveillance and reactor distributions estimated from VetNet data (Summarised in Fig. 1) conditional on our prior assumptions (Table 4). Each approximate posterior distribution is plotted on the range of the (uniform) prior distributions for each parameter. Sensitivity and specificity parameters are further constrained such that the severe interpretation always has a higher sensitivity and lower specificity (Panels A,B). On panel B a dashed vertical line indicates the lower bound of the prior distribution for specificity at the standard interpretation.
(TIF)

S3 Fig Predictive distributions for SOR model compared to persistence metrics (2003–2011). Within-herd measures of persistence and surveillance used as target metrics for ABC and to assess model fit. We present four key measures, from left to right: the proportion of prolonged (restrictions of greater duration than 240 days) and recurrent breakdowns, the proportion of herds with evidence of visible lesions and the total number of reactors per breakdown. Breakdowns are classified as either OTF-S (officially TB free suspended), where no reactors are found to have visible lesions (lime green circles), or OTF-W (officially TB free withdrawn) where at least one reactor was found to have evidence of visible lesions or be culture positive (magenta squares). The proportion of such OTF-W breakdowns is shown along with the proportion of these that were initiated by a slaughterhouse case (black circles). The relationship of each measure with herd size is plotted, with breakdowns further stratified by the historical parish testing interval (A, PTI1; B, PTI 2; C PTI 4) and breakdown status. Mean target observations are plotted with uncertainty estimated as ±1.96 standard errors around the mean. Predictive distributions from our within-herd (SOR) model for each of these measures are plotted as shaded density strips where the intensity of color is proportional to the probability density at that point [34].
(TIF)

S4 Fig SOR model predictive distributions for age of reactors. SOR model predictive distributions for the age of reactors (**A**). Age of “confirmed” reactors with evidence of visible lesions (**B**). Slaughterhouse cases (**C**) and the proportion of animals with visible lesions stratified by age (**D**). Solid points and lines indicate empirical target distributions, model predictive distributions are once again overplotted as shaded density strips where the intensity of color is proportional to the probability density at that point [34].
(TIF)

S5 Fig Break-even points for vaccine efficacy under alternative testing scenarios using γ-DIVA test (SOR model). We estimate the break-even point for a protective benefit of BCG vaccination at the herd level under three alternative testing scenarios. We model DIVA testing using parameter estimates that optimize DIVA specificity of 99.4% under the constraint of maintaining a DIVA sensitivity comparable to tuberculin testing of 64.4%. We consider four key measures of the epidemiological, and economic, costs associated with bTB testing: **A** the number of animals condemned as reactors; **B** the number of tests (tuberculin and DIVA) needed to clear restrictions; **C** The number of infected animals left in herds after restrictions are lifted (burden of infection missed by testing) **D** The number of herds that experience a breakdown before the herd clears the singleton challenge. For all panels, solid black lines indicate the median break-even point for the baseline scenario with no vaccination. Dashed lines indicate the 95% quantiles of the baseline scenario. The distribution for each measure is calculated from 100 simulations with parameters drawn from the (approximate) posterior distributions of our estimated model, with each parameter set simulated once for each herd within our representative study population (of 6,601 herds).
(TIF)

S6 Fig Probability of a protective herd level benefit of vaccination for alternative DIVA testing strategies (SOR model). We explore how the probability of seeing a protective benefit of vaccination depends on the assumed sensitivity and specificity of DIVA testing. The probability of benefit is calculated for a singleton challenge of infection and relative to the current statutory regime of tuberculin testing and slaughterhouse surveillance. Benefit is estimated from 100 simulations of our study population (6,601 herds) for three key measures (across columns): the total number of tests required to clear restrictions (**A,D,G**), the probability of restrictions being applied before the herd clears infection (**B,E,H**) and the probability of infection remaining in a herd when restrictions are lifted (**C,F,I**). We define the break-even point as 50% of herds demonstrating a protective benefit illustrated by the white band in the color map with red values worse this threshold and grey points better. We compare the three strategies described in the main text (across rows): (**A,B,C**) Under the DIVA negation scenario, the break-even point is limited by the considerable overhead in testing, with an increased probability of restrictions being applied before the herd clears infection (**B**) and an increase in testing (**A**) even for a 100% sensitive and specific DIVA test. (**D,E,F**) Under DIVA replacement, a protective benefit of vaccination can be achieved for DIVA specificities > 99.90% (**D**). The break-even point also depends on DIVA sensitivity, with a sensitivity of at least 40% being necessary to avoid increased risk of leaving infection in the herd after restrictions are lifted (**F**). (**G,H,I**) Under the VLend scenario, linking the maintenance of restrictions to detection of lesioned reactor animals mitigates the addition costs of testing under other scenarios (**G**). However, a specificity of greater than 99.85% is still required to see no increase in the number of breakdowns with vaccination (**H**), with the break-even point depending again on a DIVA sensitivity of greater than 40% (**I**).
(TIF)

S7 Fig Compartmental structure of epidemiological models. (TIF)

S8 Fig Sequence of testing before, during and after disclosure of infection in a herd. (TIF)

S9 Fig Alternative version of Fig. 4: Break-even points for vaccine efficacy under alternative testing scenarios using γ-DIVA test. We estimate the break-even point for a protective benefit of BCG vaccination at the herd level under three alternative testing scenarios. We model DIVA testing using parameter estimates that optimize DIVA specificity of 99.4% under the constraint of maintaining a DIVA sensitivity comparable to tuberculin testing of 64.4%. We consider four key measures of the epidemiological, and economic, costs associated with bTB testing: **A** the number of animals condemned as reactors; **B** the number of tests (tuberculin and DIVA) needed to clear restrictions; **C** The number of infected animals left in herds after restrictions are lifted (burden of infection missed by testing) **D** The number of herds that experience a breakdown before the herd clears the singleton challenge. For all panels, solid black lines indicate the median break-even point for the baseline scenario with no vaccination. Dashed lines indicate the 95% quantiles of the baseline scenario. The distribution for each measure is calculated from 100 simulations with parameters drawn from the (approximate) posterior distributions of our estimated model, with each parameter set simulated once for each herd within our representative study population (of 6,601 herds).
(TIF)

S10 Fig Alternative version of S5 Fig.: Break-even points for vaccine efficacy under alternative testing scenarios using γ-DIVA test (SOR model). We estimate the break-even point for a protective benefit of BCG vaccination at the herd level under three alternative testing scenarios. We model DIVA testing using parameter estimates that optimize DIVA specificity of 99.4% under the constraint of maintaining a DIVA sensitivity comparable to tuberculin testing of 64.4%. We consider four key measures of the epidemiological, and economic, costs associated with bTB testing: **A** the number of animals condemned as reactors; **B** the number of tests (tuberculin and DIVA) needed to clear restrictions; **C** The number of infected animals left in herds after restrictions are lifted (burden of infection missed by testing) **D** The number of herds that experience a breakdown before the herd clears the singleton challenge. For all panels, solid black lines indicate the median break-even point for the baseline scenario with no vaccination. Dashed lines indicate the 95% quantiles of the baseline scenario. The distribution for each measure is calculated from 100 simulations with parameters drawn from the (approximate) posterior distributions of our estimated model, with each parameter set simulated once for each herd within our representative study population (of 6,601 herds).
(TIF)

S1 Dataset Data tables defining herd demography and target epidemiological metrics. (ZIP)

## References

[1] Waters WR, Palmer MV, Buddle BM, Vordermeier HM (2012) Bovine tuberculosis vaccine research: Historical perspectives and recent advances. Vaccine 30: 2611–2622. 10.1016/j.vaccine.2012.02.018 22342705

[2] Hope JC, Thom ML, Villarreal-Ramos B, Vordermeier HM, Hewinson RG, et al (2005) Vaccination of neonatal calves with Mycobacterium bovis BCG induces protection against intranasal challenge with virulent M. bovis. Clin Exp Immunol 139: 48–56. 15606613 10.1111/j.1365-2249.2005.02668.x PMC1809274

[3] Suazo FM, Escalera AMA, Torres RMG (2003) A review of M. bovis BCG protection against TB in cattle and other animals species. Prev Vet Med 58: 1–13. 12628767 10.1016/s0167-5877(03)00003-5

[4] Lopez-Valencia G, Renteria-Evangelista T, de J. Williams J, Licea-Navarro A, la Mora-Valle AD, et al (2010) Field evaluation of the protective efficacy of Mycobacterium bovis BCG vaccine against bovine tuberculosis. Res Vet Sci 88: 44–49. 10.1016/j.rvsc.2009.05.022 19564029

[5] Ameni G, Vordermeier M, Aseffa A, Young DB, Hewinson RG (2010) Field Evaluation of the Efficacy of Mycobacterium bovis Bacillus Calmette-Guérin against Bovine Tuberculosis in Neonatal Calves in Ethiopia. Clin Vaccine Immunol 17: 1533–1538. 10.1128/CVI.00222-10 20719984 PMC2953002

[6] Whelan AO, Coad M, Upadhyay BL, Clifford DJ, Hewinson RG, et al (2011) Lack of correlation between BCG-induced tuberculin skin test sensitisation and protective immunity in cattle. Vaccine 29: 5453–5458. 10.1016/j.vaccine.2011.05.057 21640776

[7] Conlan AJK, McKinley TJ, Karolemeas K, Brooks Pollock E, Goodchild AV, et al (2012) Estimating the Hidden Burden of Bovine Tuberculosis in Great Britain. PLoS Comput Biol 8: e1002730 10.1371/journal.pcbi.1002730 23093923 PMC3475695

[8] Vordermeier M S. V. Gordon SV, Hewinson RG (2011) Mycobacterium bovis antigens for the differential diagnosis of vaccinated and infected cattle. Vet. Microbiol 151: 8–13. 10.1016/j.vetmic.2011.02.020 21411245

[9] Jones GJ, Whelan A, Clifford D, Coad M, Vordermeier HM (2012) Improved Skin Test for Differential Diagnosis of Bovine Tuberculosis by the Addition of Rv3020c-Derived Peptides. Clin Vaccine Immunol 19: 620–622. 10.1128/CVI.00024-12 22301696 PMC3318272

[10] Goodchild AV, Clifton-Hadley RS (2001) Cattle-to-cattle transmission of Mycobacterium bovis. Tuberculosis 81: 23–41. 11463222 10.1054/tube.2000.0256

[11] de la Rua-Domenech R, Goodchild AT, Vordermeier HM, Hewinson RG, Christiansen KH, et al (2006) Ante mortem diagnosis of tuberculosis in cattle: A review of the tuberculin tests, γ-interferon assay and other ancillary diagnostic techniques. Res Vet Sci 81: 190–210. 16513150 10.1016/j.rvsc.2005.11.005

[12] Karolemeas K, de la Rua-Domenech R, Cooper R, Goodchild AV, Clifton-Hadley RS, et al (2012) Estimation of the Relative Sensitivity of the Comparative Tuberculin Skin Test in Tuberculous Cattle Herds Subjected to Depopulation. PLoS ONE 7:e43217 10.1371/journal.pone.0043217 22927952 PMC3424237

[13] EFSA (2012) Scientific Opinion on the use of a gamma interferon test for the diagnosis of bovine tuberculosis. EFSA J 10: 2975.

[14] Karolemeas K, McKinley TJ, Clifton-Hadley RS, Goodchild AV, Mitchell A, et al (2010) Predicting prolonged bovine tuberculosis breakdowns in Great Britain as an aid to control. Prev Vet Med 97: 183–190. 10.1016/j.prevetmed.2010.09.007 20965599

[15] Bennett RM, Cooke RJ (2006) Costs to farmers of a tuberculosis breakdown. Vet Rec 158:429–432. 16581992 10.1136/vr.158.13.429

[16] Abernethy DA, Upton P, Higgins IM, McGrath G, Goodchild AV, et al, (2013) Bovine tuberculosis trends in the UK and the Republic of Ireland, 1995–2010. Vet Rec 172: 312–312. 10.1136/vr.100969 23292950

[17] Godfray HCJ, Donnelly CA, Kao RR, Macdonald DW, McDonald RA, et al (2013) A restatement of the natural science evidence base relevant to the control of bovine tuberculosis in Great Britain. Proc R Soc B Biol Sci 280:20131634 10.1098/rspb.2013.1634 23926157 PMC3757986

[18] Anderson R and May R (1991) Infectious Diseases of Humans: Dynamics and Control. Oxford University Press, New York 768 p.

[19] EFSA (2013) Scientific Opinion on field trials for bovine tuberculosis vaccination. EFSA J 11: 3475.

[20] Bennett R, Balcombe K (2012) Farmers’ Willingness to Pay for a Tuberculosis Cattle Vaccine. J Agric Econ 63: 408–424.

[21] Brooks-Pollock E E., Conlan AJ, Mitchell AP, Blackwell R, McKinley TJ, et al (2013) Age-dependent patterns of bovine tuberculosis in cattle. Vet Res 44: 97 10.1186/1297-9716-44-97 24131703 PMC3853322

[22] Barlow ND, Kean JM, Hickling G, Livingstone PG, Robson AB (1997) A simulation model for the spread of bovine tuberculosis within New Zealand cattle herds. Prev Vet Med 32: 57–75. 9361321 10.1016/s0167-5877(97)00002-0

[23] Kao RR, Roberts MG, Ryan TJ (1997) A model of bovine tuberculosis control in domesticated cattle herds. Proc R Soc Lond B Biol Sci 264: 1069–1076. 10.1098/rspb.1997.0148 PMC1688544 9263472

[24] Fischer EAJ, van Roermund HJW, Hemerik L, van Asseldonk MAPM, de Jong MCM (2005) Evaluation of surveillance strategies for bovine tuberculosis (Mycobacterium bovis) using an individual based epidemiological model. Prev Vet Med 67: 283–301. 15748757 10.1016/j.prevetmed.2004.12.002

[25] Kao RR, Gravenor MB, Charleston B, Hope JC, Martin M, et al (2007) Mycobacterium bovis shedding patterns from experimentally infected calves and the effect of concurrent infection with bovine viral diarrhoea virus. J. R. Soc. Interface 4: 545–551. 17251130 10.1098/rsif.2006.0190 PMC1871617

[26] Toni T, Welch D, Strelkowa M, Ipsen A, Stumpf MPH (2009) Approximate Bayesian computation scheme for parameter inference and model selection in dynamical systems. J R Soc Interface 9: 187–202. 10.1098/rsif.2008.0172 PMC2658655 19205079

[27] Shittu A, Clifton-Hadley RS, Ely ER, Upton PU, Downs SH (2013) Factors associated with bovine tuberculosis confirmation rates in suspect lesions found in cattle at routine slaughter in Great Britain, 2003–2008. Prev Vet Med 110: 395–404. 10.1016/j.prevetmed.2013.03.001 23540447

[28] Enticott G (2012) The local universality of veterinary expertise and the geography of animal disease Trans. Inst. Br. Geogr 37: 75–88.

[29] Gutenkunst RN, Waterfall JJ, Casey FP, Brown KS, Myers CR, et al (2007) Universally Sloppy Parameter Sensitivities in Systems Biology Models. PLoS Comput Biol 3:e189. 10.1371/journal.pcbi.0030189 PMC2000971 17922568

[30] Pai M, Zwerling A, Menzies D (2008) Systematic Review: T-Cell—based Assays for the Diagnosis of Latent Tuberculosis Infection: An Update. Ann Intern Med 149: 177–184. 18593687 10.7326/0003-4819-149-3-200808050-00241 PMC2951987

[31] Marjoram P, Molitor J, Plagnol V, Tavaré S (2003) Markov chain Monte Carlo without likelihoods. PNAS 100: 15324–15328. 14663152 10.1073/pnas.0306899100 PMC307566

[32] Gillespie D (1976) A general method for numerically simulating the stochastic time evolution of couple chemical reactions. J Comput Phys 22: 403–434.

[33] Hope JC, Thom ML, McAulay M, Mead E, Vordermeier HM, et al (2011) Identification of Surrogates and Correlates of Protection in Protective Immunity against Mycobacterium bovis Infection Induced in Neonatal Calves by Vaccination with M. bovis BCG Pasteur and M. bovis BCG Danish. Clin Vaccine Immunol 18: 373–379. 10.1128/CVI.00543-10 21228141 PMC3067386

[34] Jackson CH (2008) Displaying uncertainty with shading. Am Stat, 62: 340–347.

