# Supplementary figures and images for: Potential Benefits of Cattle Vaccination as a Supplementary Control for Bovine Tuberculosis
Source: PLoS Comput Biol. 2015 Feb 19;11(2):e1004038. doi: 10.1371/journal.pcbi.1004038 (PMC4335026; doi:10.1371/journal.pcbi.1004038)

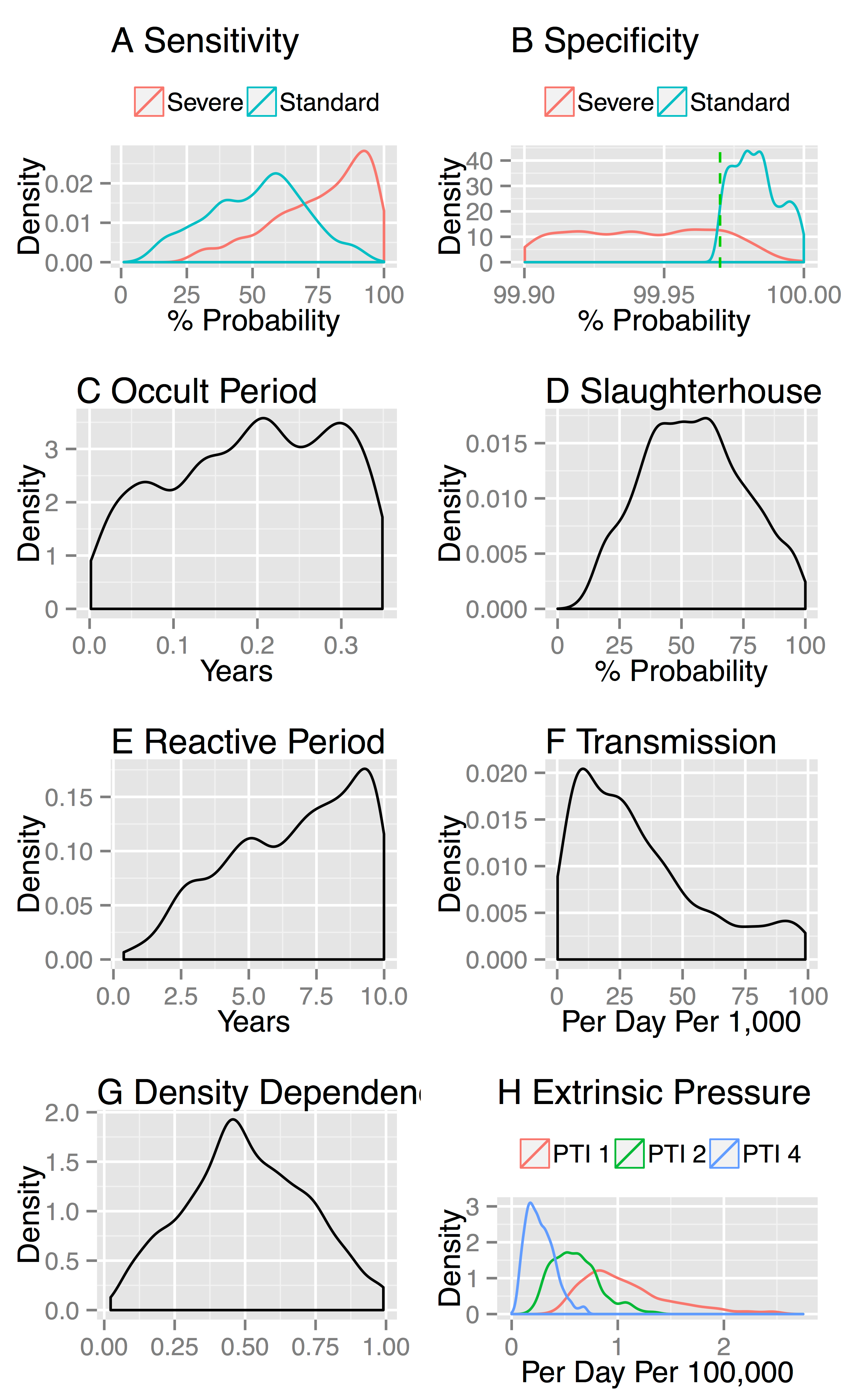

Supplement: S1 Fig — Parameter distributions estimated for SORI model by ABC-SMC. Distributions of parameters most consistent with persistence, surveillance and reactor distributions estimated from VetNet data (Summarised in Fig. 1) conditional on our prior assumptions (Table 4). Each approximate posterior distribution is plotted on the range of the (uniform) prior distributions for each parameter. Sensitivity and specificity parameters are further constrained such that the severe interpretation always has a higher sensitivity and lower specificity (Panels A,B). On panel B a dashed vertical line indicates the lower bound of the prior distribution for specificity at the standard interpretation. (TIF) [file pcbi.1004038.s001.tif]

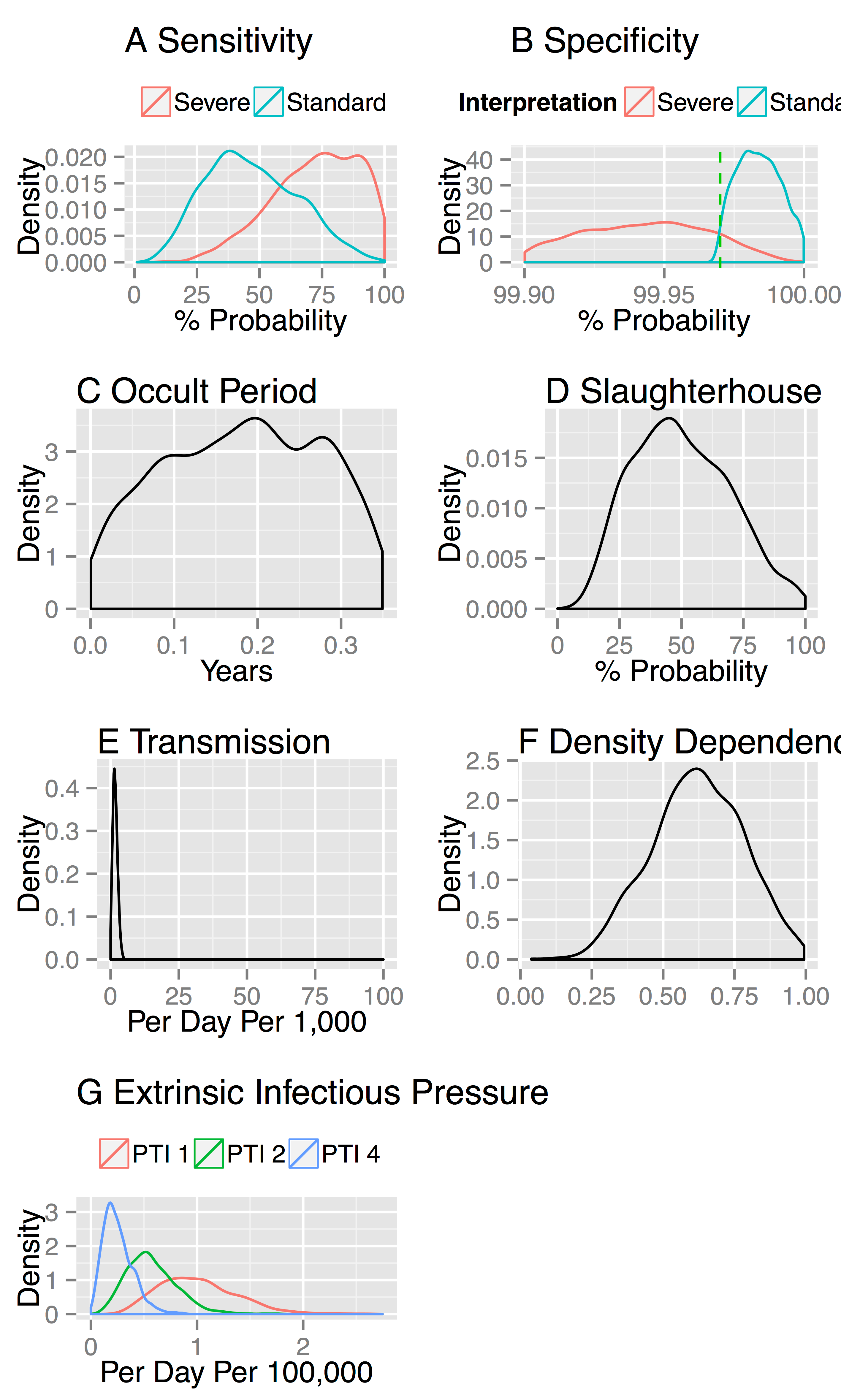

Supplement: S2 Fig — Parameter distributions estimated for SOR model by ABC-SMC. Distributions of parameters most consistent with persistence, surveillance and reactor distributions estimated from VetNet data (Summarised in Fig. 1) conditional on our prior assumptions (Table 4). Each approximate posterior distribution is plotted on the range of the (uniform) prior distributions for each parameter. Sensitivity and specificity parameters are further constrained such that the severe interpretation always has a higher sensitivity and lower specificity (Panels A,B). On panel B a dashed vertical line indicates the lower bound of the prior distribution for specificity at the standard interpretation. (TIF) [file pcbi.1004038.s002.tif]

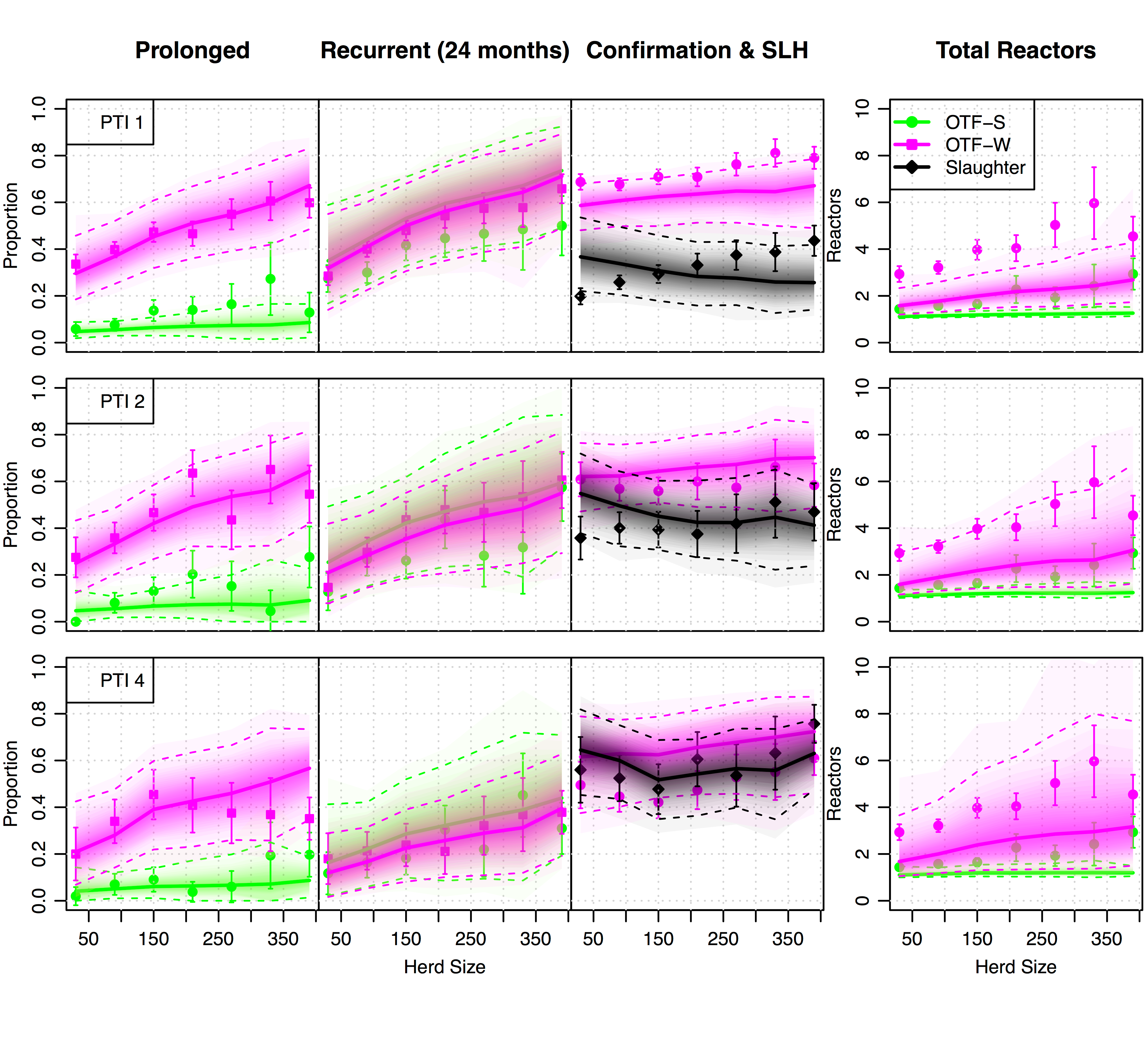

Supplement: S3 Fig — Within-herd measures of persistence and surveillance used as target metrics for ABC and to assess model fit. We present four key measures, from left to right: the proportion of prolonged (restrictions of greater duration than 240 days) and recurrent breakdowns, the proportion of herds with evidence of visible lesions and the total number of reactors per breakdown. Breakdowns are classified as either OTF-S (officially TB free suspended), where no reactors are found to have visible lesions (lime green circles), or OTF-W (officially TB free withdrawn) where at least one reactor was found to have evidence of visible lesions or be culture positive (magenta squares). The proportion of such OTF-W breakdowns is shown along with the proportion of these that were initiated by a slaughterhouse case (black circles). The relationship of each measure with herd size is plotted, with breakdowns further stratified by the historical parish testing interval (A, PTI1; B, PTI 2; C PTI 4) and breakdown status. Mean target observations are plotted with uncertainty estimated as ±1.96 standard errors around the mean. Predictive distributions from our within-herd (SOR) model for each of these measures are plotted as shaded density strips where the intensity of color is proportional to the probability density at that point [34]. (TIF) [file pcbi.1004038.s003.tif]

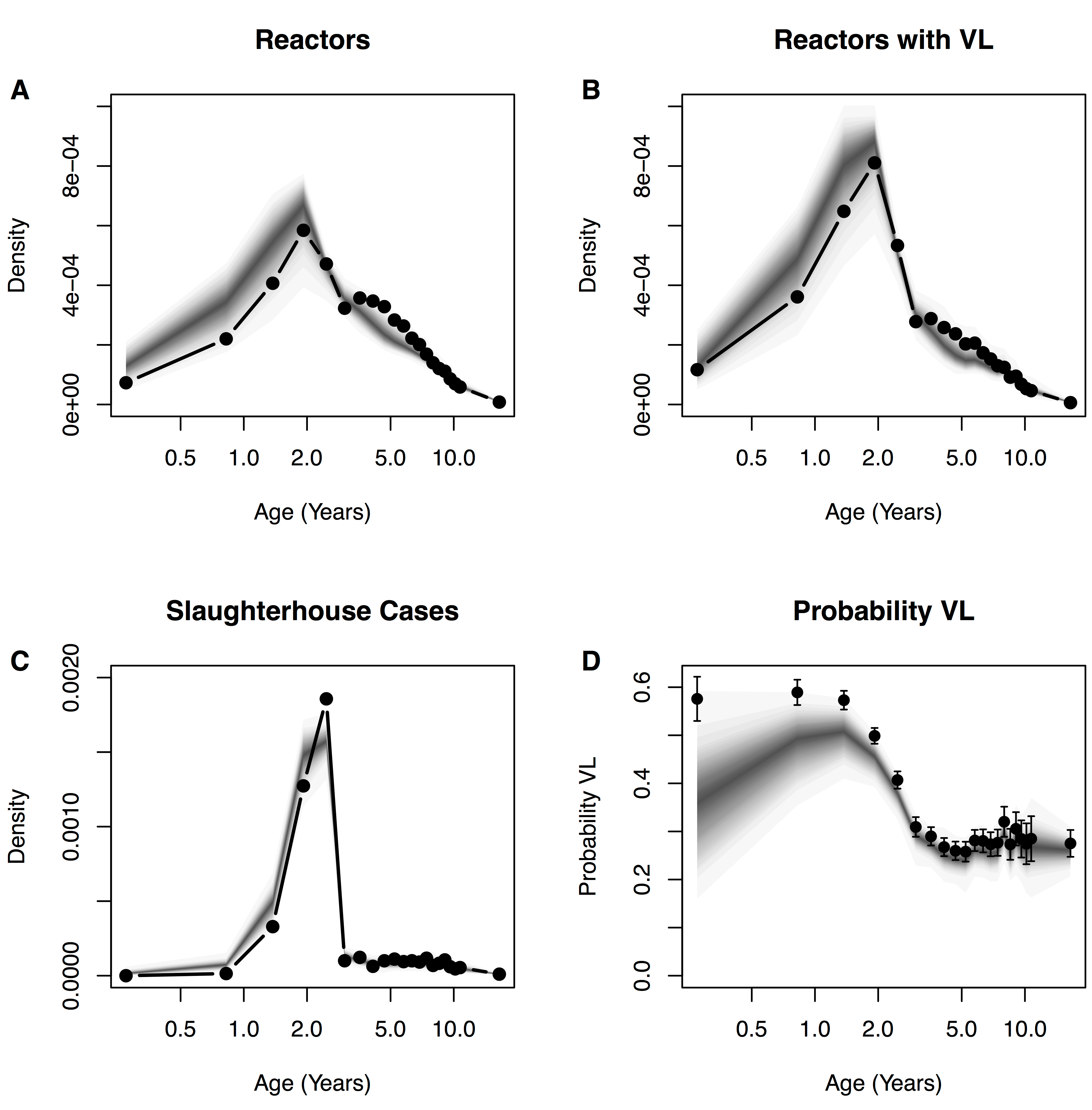

Supplement: S4 Fig — SOR model predictive distributions for the age of reactors (A). Age of “confirmed” reactors with evidence of visible lesions (B). Slaughterhouse cases (C) and the proportion of animals with visible lesions stratified by age (D). Solid points and lines indicate empirical target distributions, model predictive distributions are once again overplotted as shaded density strips where the intensity of color is proportional to the probability density at that point [34]. (TIF) [file pcbi.1004038.s004.tif]

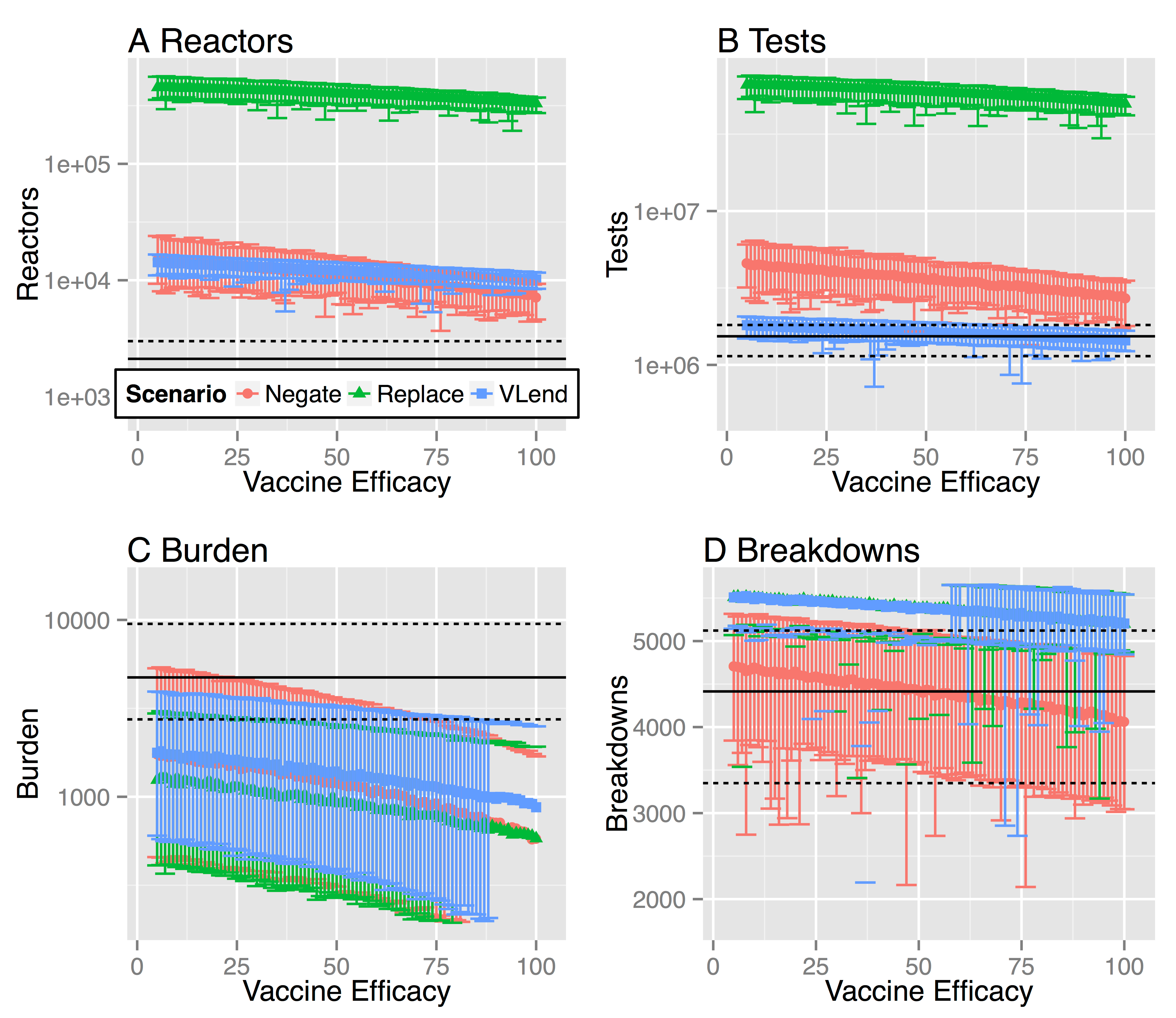

Supplement: S5 Fig — We estimate the break-even point for a protective benefit of BCG vaccination at the herd level under three alternative testing scenarios. We model DIVA testing using parameter estimates that optimize DIVA specificity of 99.4% under the constraint of maintaining a DIVA sensitivity comparable to tuberculin testing of 64.4%. We consider four key measures of the epidemiological, and economic, costs associated with bTB testing: A the number of animals condemned as reactors; B the number of tests (tuberculin and DIVA) needed to clear restrictions; C The number of infected animals left in herds after restrictions are lifted (burden of infection missed by testing) D The number of herds that experience a breakdown before the herd clears the singleton challenge. For all panels, solid black lines indicate the median break-even point for the baseline scenario with no vaccination. Dashed lines indicate the 95% quantiles of the baseline scenario. The distribution for each measure is calculated from 100 simulations with parameters drawn from the (approximate) posterior distributions of our estimated model, with each parameter set simulated once for each herd within our representative study population (of 6,601 herds). (TIF) [file pcbi.1004038.s005.tif]

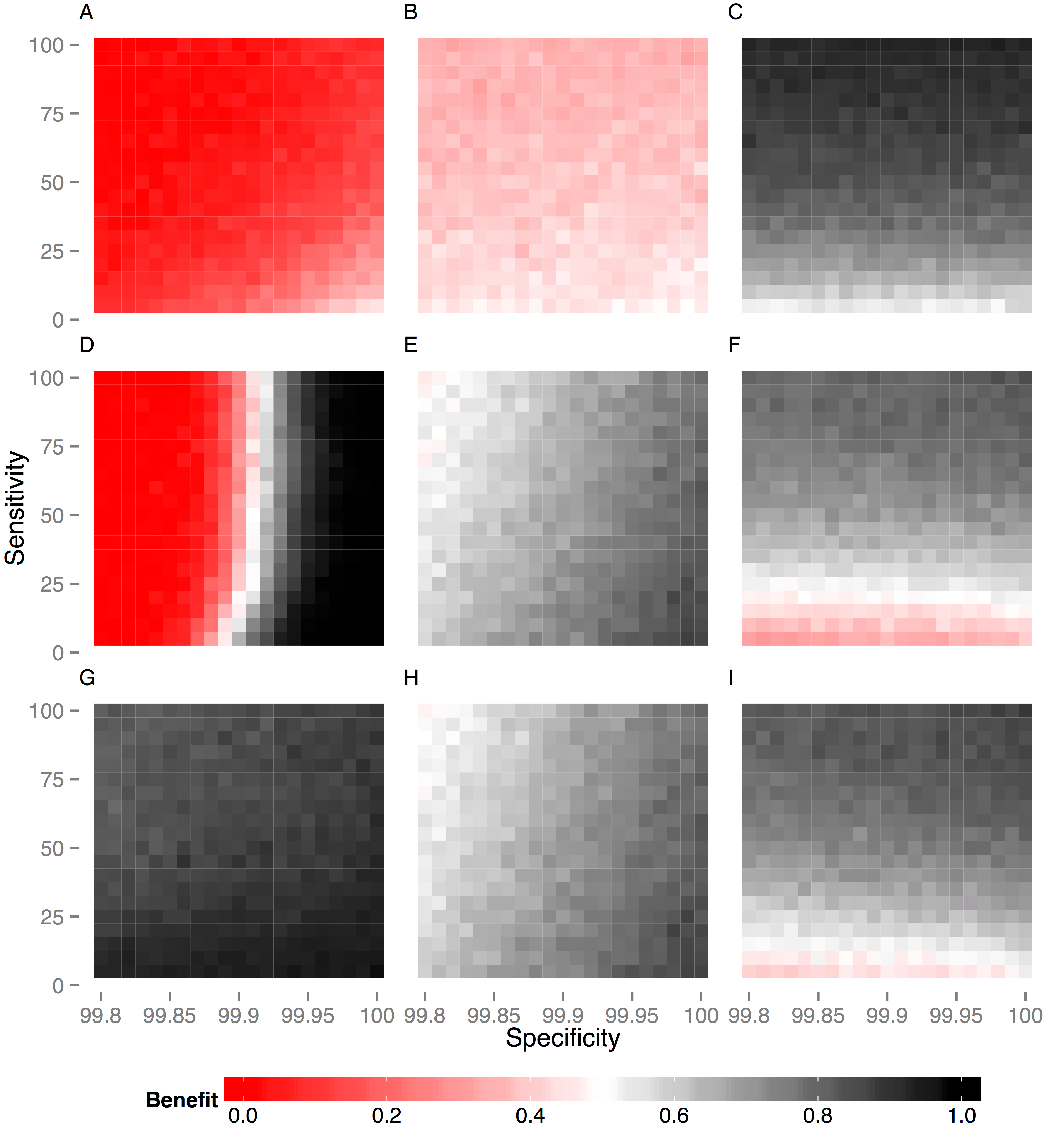

Supplement: S6 Fig — We explore how the probability of seeing a protective benefit of vaccination depends on the assumed sensitivity and specificity of DIVA testing. The probability of benefit is calculated for a singleton challenge of infection and relative to the current statutory regime of tuberculin testing and slaughterhouse surveillance. Benefit is estimated from 100 simulations of our study population (6,601 herds) for three key measures (across columns): the total number of tests required to clear restrictions (A,D,G), the probability of restrictions being applied before the herd clears infection (B,E,H) and the probability of infection remaining in a herd when restrictions are lifted (C,F,I). We define the break-even point as 50% of herds demonstrating a protective benefit illustrated by the white band in the color map with red values worse this threshold and grey points better. We compare the three strategies described in the main text (across rows): (A,B,C) Under the DIVA negation scenario, the break-even point is limited by the considerable overhead in testing, with an increased probability of restrictions being applied before the herd clears infection (B) and an increase in testing (A) even for a 100% sensitive and specific DIVA test. (D,E,F) Under DIVA replacement, a protective benefit of vaccination can be achieved for DIVA specificities > 99.90% (D). The break-even point also depends on DIVA sensitivity, with a sensitivity of at least 40% being necessary to avoid increased risk of leaving infection in the herd after restrictions are lifted (F). (G,H,I) Under the VLend scenario, linking the maintenance of restrictions to detection of lesioned reactor animals mitigates the addition costs of testing under other scenarios (G). However, a specificity of greater than 99.85% is still required to see no increase in the number of breakdowns with vaccination (H), with the break-even point depending again on a DIVA sensitivity of greater than 40% (I). (TIF) [file pcbi.1004038.s006.tif]

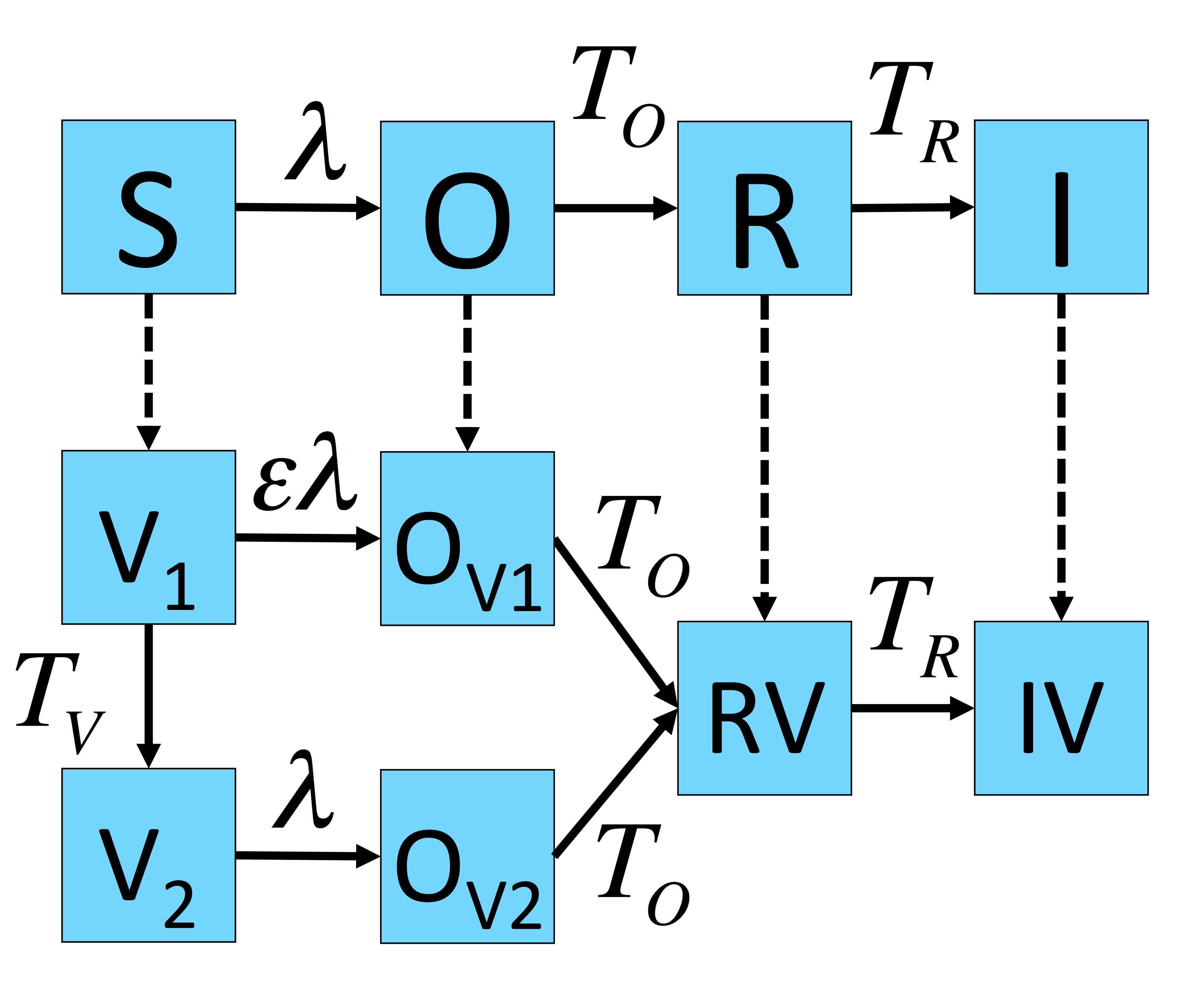

Supplement: S7 Fig — (TIF) [file pcbi.1004038.s007.tif]

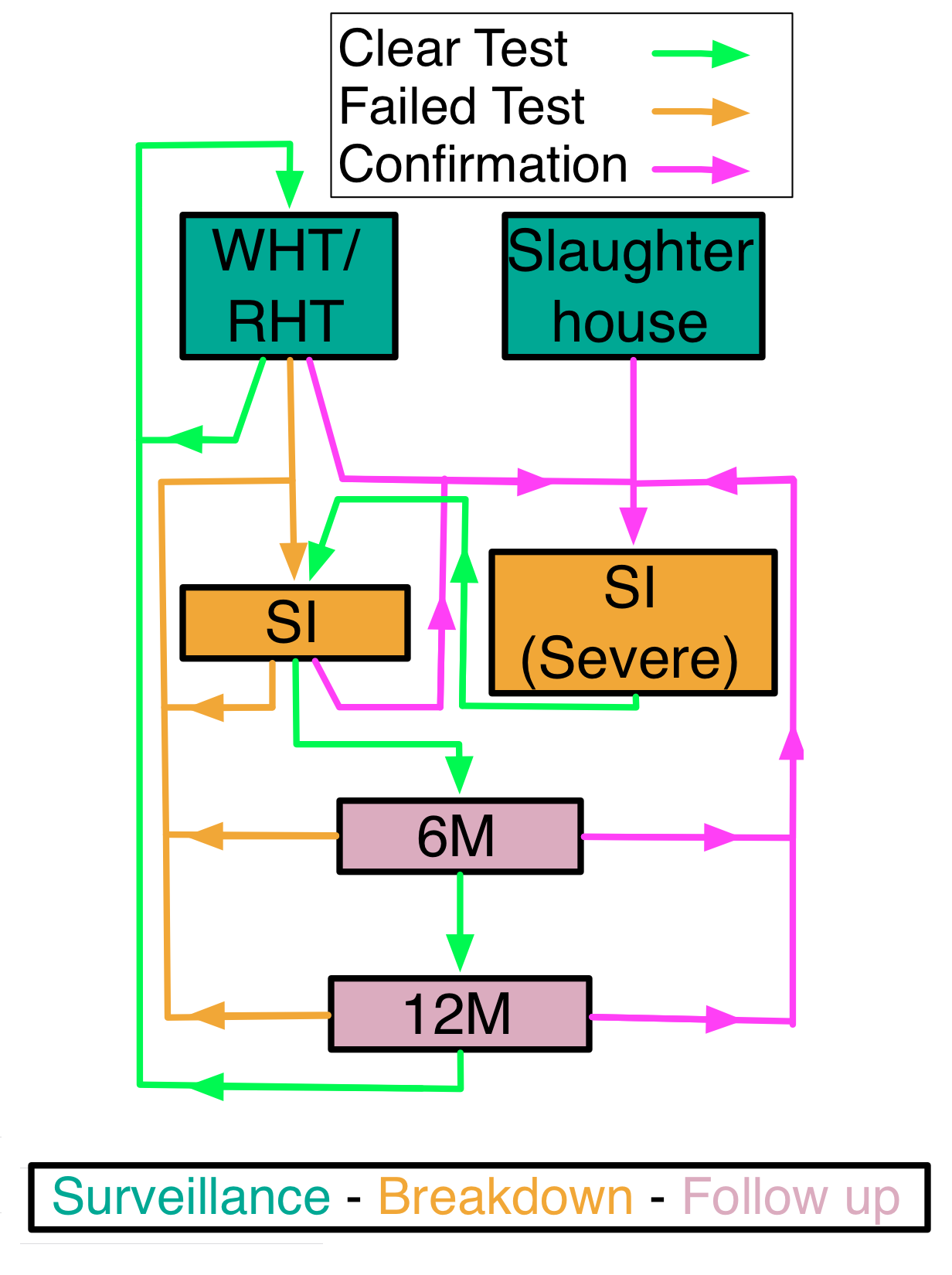

Supplement: S8 Fig — (TIF) [file pcbi.1004038.s008.tif]

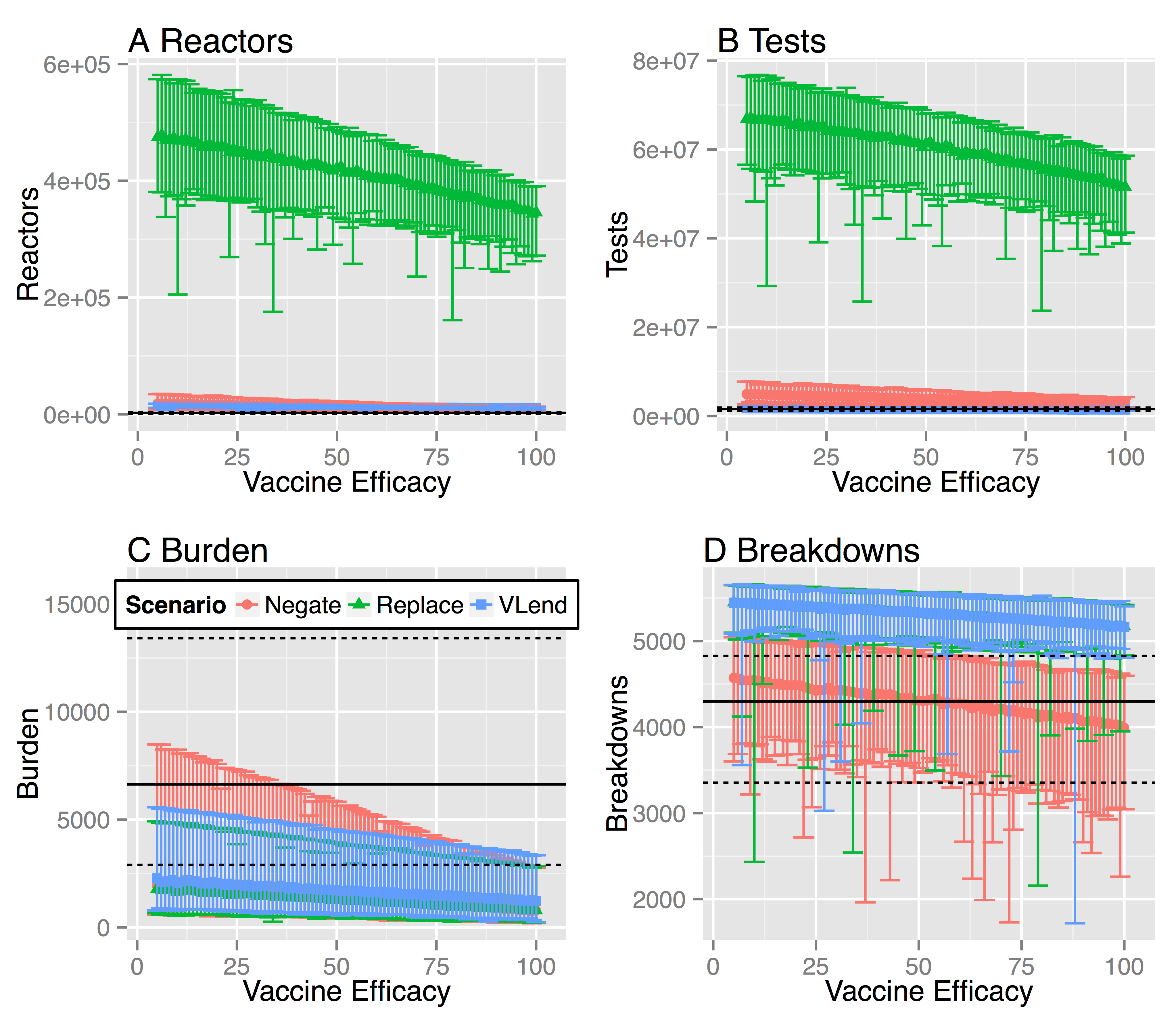

Supplement: S9 Fig — We estimate the break-even point for a protective benefit of BCG vaccination at the herd level under three alternative testing scenarios. We model DIVA testing using parameter estimates that optimize DIVA specificity of 99.4% under the constraint of maintaining a DIVA sensitivity comparable to tuberculin testing of 64.4%. We consider four key measures of the epidemiological, and economic, costs associated with bTB testing: A the number of animals condemned as reactors; B the number of tests (tuberculin and DIVA) needed to clear restrictions; C The number of infected animals left in herds after restrictions are lifted (burden of infection missed by testing) D The number of herds that experience a breakdown before the herd clears the singleton challenge. For all panels, solid black lines indicate the median break-even point for the baseline scenario with no vaccination. Dashed lines indicate the 95% quantiles of the baseline scenario. The distribution for each measure is calculated from 100 simulations with parameters drawn from the (approximate) posterior distributions of our estimated model, with each parameter set simulated once for each herd within our representative study population (of 6,601 herds). (TIF) [file pcbi.1004038.s009.tif]

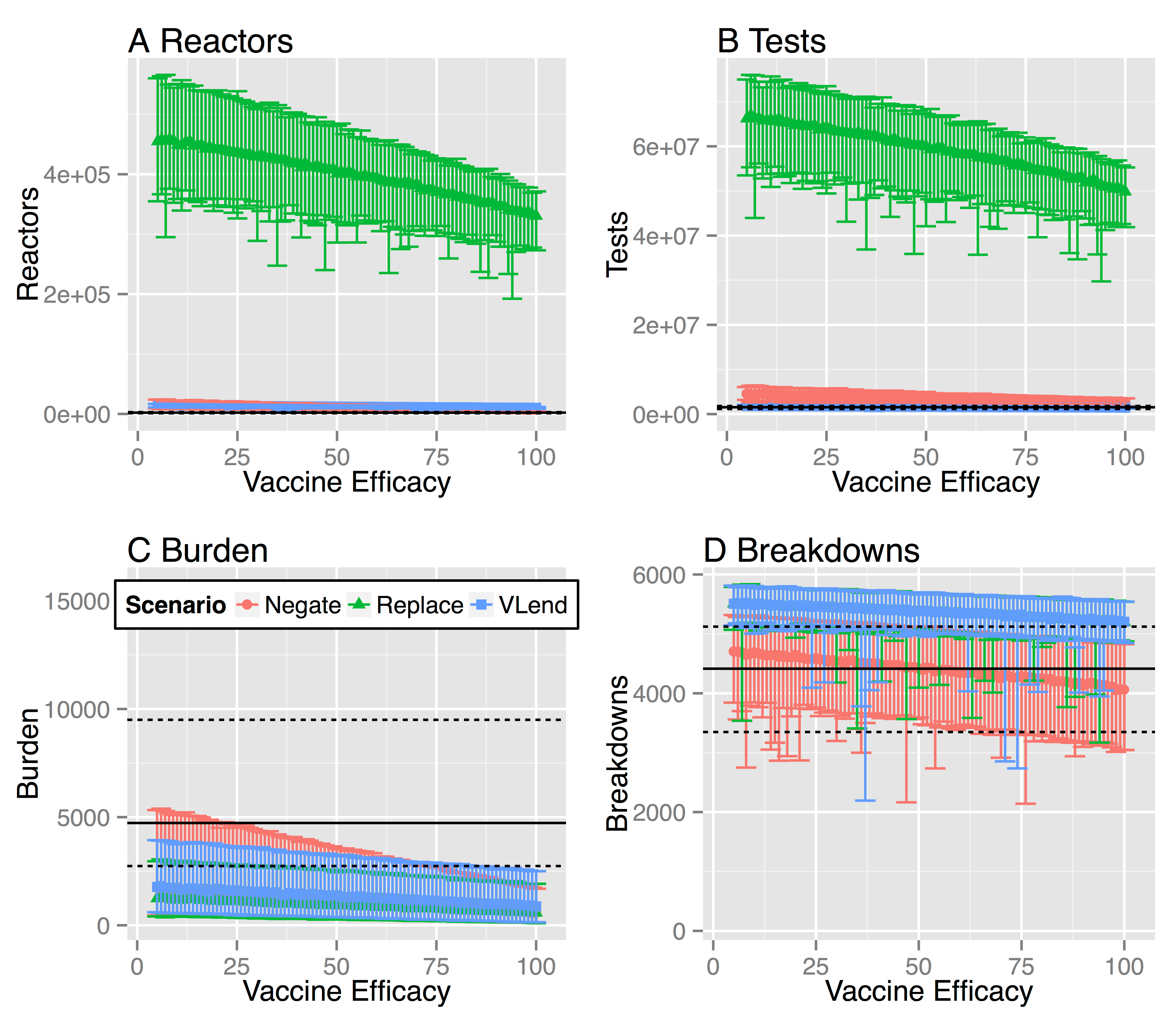

Supplement: S10 Fig — We estimate the break-even point for a protective benefit of BCG vaccination at the herd level under three alternative testing scenarios. We model DIVA testing using parameter estimates that optimize DIVA specificity of 99.4% under the constraint of maintaining a DIVA sensitivity comparable to tuberculin testing of 64.4%. We consider four key measures of the epidemiological, and economic, costs associated with bTB testing: A the number of animals condemned as reactors; B the number of tests (tuberculin and DIVA) needed to clear restrictions; C The number of infected animals left in herds after restrictions are lifted (burden of infection missed by testing) D The number of herds that experience a breakdown before the herd clears the singleton challenge. For all panels, solid black lines indicate the median break-even point for the baseline scenario with no vaccination. Dashed lines indicate the 95% quantiles of the baseline scenario. The distribution for each measure is calculated from 100 simulations with parameters drawn from the (approximate) posterior distributions of our estimated model, with each parameter set simulated once for each herd within our representative study population (of 6,601 herds). (TIF) [file pcbi.1004038.s010.tif]
